# Expansion of functional personalized cells with specific transgene combinations

**DOI:** 10.1038/s41467-018-03408-4

**Published:** 2018-03-08

**Authors:** Christoph Lipps, Franziska Klein, Tom Wahlicht, Virginia Seiffert, Milada Butueva, Jeannette Zauers, Theresa Truschel, Martin Luckner, Mario Köster, Roderick MacLeod, Jörn Pezoldt, Jochen Hühn, Qinggong Yuan, Peter Paul Müller, Henning Kempf, Robert Zweigerdt, Oliver Dittrich-Breiholz, Thomas Pufe, Rainer Beckmann, Wolf Drescher, Jose Riancho, Carolina Sañudo, Thomas Korff, Bertram Opalka, Vera Rebmann, Joachim R. Göthert, Paula M. Alves, Michael Ott, Roland Schucht, Hansjörg Hauser, Dagmar Wirth, Tobias May

**Affiliations:** 1 Model Systems for Infection and Immunity, HZI – Helmholtz Centre for Infection Research, Inhoffenstr. 7, 38124 Braunschweig, Germany; 2 Department of Gene Regulation and Differentiation, HZI – Helmholtz Centre for Infection Research, Inhoffenstr. 7, 38124 Braunschweig, Germany; 3 InSCREENeX GmbH, Inhoffenstr. 7, 38124 Braunschweig, Germany; 4 Leibniz Institute DSMZ – German Collection of Microorganisms and Cell Cultures, Inhoffenstr. 7, 38124 Braunschweig, Germany; 5 Experimental Immunology, HZI – Helmholtz Centre for Infection Research, Inhoffenstr. 7, 38124 Braunschweig, Germany; 6 Department of Gastroenterology, Hepatology, Endocrinology, Hannover Medical School, Carl-Neuberg-Str. 1, 30625 Hannover, Germany; 7 Translational Research Group Cell and Gene Therapy, Twincore - Centre for Experimental and Clinical Infection Research GmbH, Feodor-Lynen-Str. 7, 30625 Hannover, Germany; 8 Leibniz Research Laboratories for Biotechnology and Artificial Organs (LEBAO), Hannover Medical School, MHH, Carl-Neuberg-Str. 1, 30625 Hannover, Germany; 9 Research Core Unit Genomics, Medical School Hannover, 30625 Hannover, Germany; 10 Department of Anatomy and Cell Biology, RWTH Aachen University, 52074 Aachen, Germany; 11 Department of Orthopaedics, Aachen University Hospital, RWTH Aachen University, Aachen, 52074 Germany; 12 Department of Orthopedic Surgery of the Lower Limb and Arthroplasty, Rummelsberg Hospital, Schwarzenbruck, 90592 Germany; 13 Department of Internal Medicine, Hospital U.M. Valdecilla, University of Cantabria, IDIVAL, 39008 Santander, Spain; 14 Institute of Physiology and Pathophysiology, RG Blood Vessel Remodeling, University Heidelberg, Im Neuenheimer Feld 326, 69120 Heidelberg, Germany; 15 Department of Hematology, West German Cancer Center (WTZ), University Hospital Essen, Hufelandstr. 55, 45147 Essen, Germany; 16 Institute for Transfusion Medicine, University Hospital Essen, Virchowstr. 179, 45147 Essen, Germany; 17 Instituto de Biologia Experimental e Tecnologica, Universidade Nova de Lisboa, Oeiras, 2781-901 Portugal; 18 Experimental Hematology, Hannover Medical School, Carl-Neuberg-Str. 1, 30625 Hannover, Germany; 19 Present Address: Experimental Cardiology, Justus-Liebig University Giessen, Aulweg 129, 35392 Giessen, Germany

## Abstract

Fundamental research and drug development for personalized medicine necessitates cell cultures from defined genetic backgrounds. However, providing sufficient numbers of authentic cells from individuals poses a challenge. Here, we present a new strategy for rapid cell expansion that overcomes current limitations. Using a small gene library, we expanded primary cells from different tissues, donors, and species. Cell-type-specific regimens that allow the reproducible creation of cell lines were identified. In depth characterization of a series of endothelial and hepatocytic cell lines confirmed phenotypic stability and functionality. Applying this technology enables rapid, efficient, and reliable production of unlimited numbers of personalized cells. As such, these cell systems support mechanistic studies, epidemiological research, and tailored drug development.

## Abstract (web summary)

Personalised medicine requires cell cultures from defined genetic backgrounds, but providing sufficient numbers of cells is a challenge. Here the authors develop gene cocktails to expand primary cells from a variety of different tissues and species, and show that expanded endothelial and hepatic cells retain properties of the differentiated phenotype.

## Introduction

Cell culture is an essential tool to study the fundamentals of genetic background variables. With the development of personalized medicine, this applies increasingly to the development and safety testing of drugs. Currently, primary cells are used for these purposes. However, primary cells are usually not available in sufficient numbers and the reproducibility of assays is limited. The induced-pluripotent stem (iPS) cell technology provides access to virtually any cell type of individuals by in vitro differentiation of iPS cells, reviewed in^1,2^. Transdifferentiation or direct reprogramming of terminally differentiated cells has also been used to generate various cell types^3,4^ (reviewed in^5–7^). However, these techniques generate heterogeneous cell populations. More importantly, such approaches are limited by the fact that iPS cell-derived, terminally differentiated cells typically show no or low proliferative capacity and do not allow cell expansion^8^. Thus, methods for the rapid, efficient, and reproducible creation of expandable and authentic, i.e., physiological cell systems are required.

Transgene-driven immortalization represents an attractive option for cell expansion^9,10^. These approaches usually rely on the expression of viral oncogenes like SV40 large T antigen (*TAg*), *E6/E7* from the human papilloma virus, or *E1A/E1B* from adenovirus. Achieving indefinite proliferation requires the viral oncogenes to be highly expressed which in turn leads to an alteration of the cellular phenotype and is often accompanied by chromosomal instability; thereby, limiting the use of such cell lines (reviewed in^11,12^). The cellular gene encoding human telomerase reverse transcriptase (*hTert*) has been successfully used for the expansion of several cell types that retain important properties in vitro^13–15^. However, this approach is restricted to certain cell types as others either need the concerted action of additional immortalizing genes or the inactivation of tumor suppressor genes^16–19^.

Several reports have demonstrated that transgene-driven immortalization approaches can result in expansion of cell lines that closely reflect their in vivo counterparts^20–24^. However, respective protocols have not been explored in a way that they can be applied to other cell types. To develop a generally applicable tool for the creation of authentic cell lines we employed a library of transgenes that leads to sustained proliferation and unlimited cell expansion of different primary cells. By defining sets of genes for specific cell types, a reproducible approach was developed to derive and expand donor- and tissue-specific cell cultures. Using a series of cell lines derived from endothelium and liver parenchyma, we demonstrate that such cell lines retain the phenotypic properties and functions of their primary cell precursors. The strategy represents a widely applicable method for unlimited expansion of cells from various species and tissues.

## Results

### Unlimited expansion of primary cells with 33 selected genes

**Fig. 1:**
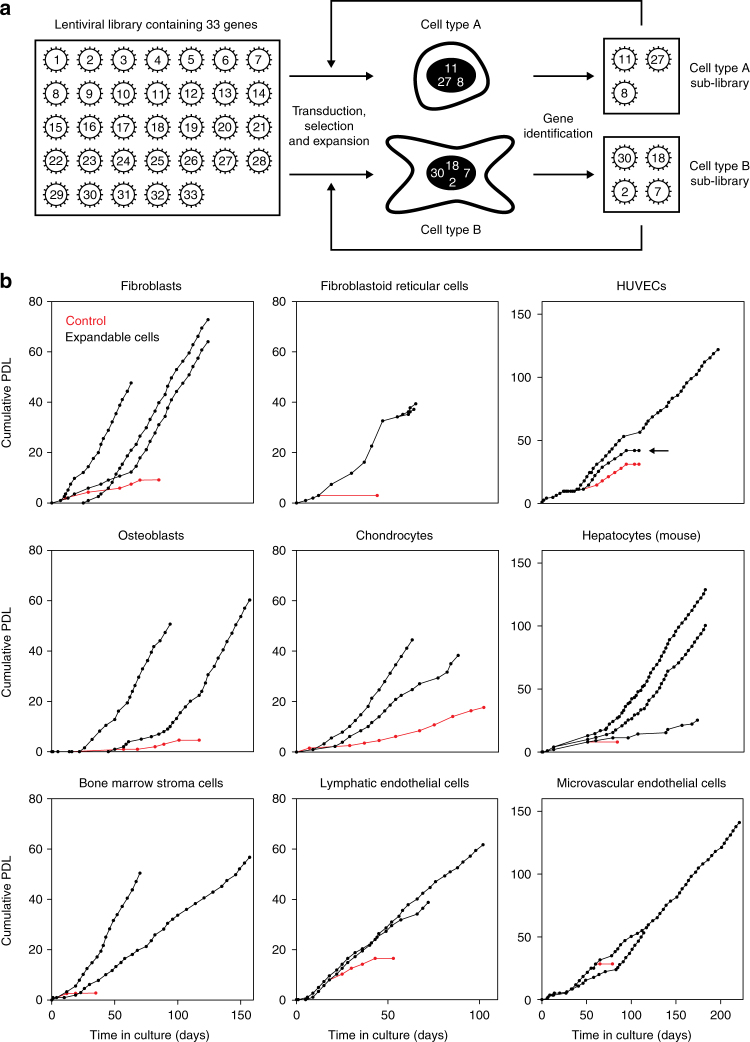
Overall strategy and proliferation capacity of transduced cells. **a** Outline of the experimental strategy followed to establish cell lines. **b** Growth curves of various human and murine cell populations upon lentiviral transduction with the library of 33 genes (black lines), as well as of mock-infected primary cells (control, red lines). Cumulative population doubling levels (PDL) of the established cell lines as well as of respective primary cells are given for the indicated time period after infection (day 0). Transducing HUVECs with a single gene (MYC) resulted in limited expansion of the cells (marked by an arrow)

For expansion of primary cells, we employed a lentiviral vector library encoding 33 genes. These genes target various cellular processes like cell-cycle progression, apoptosis, maintaining stem cell properties, or are known to achieve immortalization in specific settings (Supplementary Table 1). This library was used to randomly infect primary cultures of different cell types from various juvenile or adult human donors, as well as from wildtype or transgenic mice (Fig. 1a). Long-term expandable cell cultures were established from all tested cell types including chondrocytes, epithelial, and endothelial cells and hepatocytes showing the wide applicability of the library for immortalization of diverse cell types (for an overview of the various cell types immortalized in this study see Table 1).

**Table 1:** Overview of cell types immortalized in this study

Cell type	Name	Integrated genes	exp.	Tumor (s.c.)	Tumor (liver)
Human chondrocytes	e-hChon-1	*ID2*, *FOS*, *TAg*, *ID3*, *E7*, *E6*, *Bmi1*, *PymT*, *Core*, *Klf4*, *ID1*, *MYC*, *Nanog*, *EZH2*	p		
	e-hChon-2	*ID2*, *FOS*, *ID3*, *E7*, *Core*, *Klf4*, *ID1*, *Lmo2*, *Nanog*, *SOX2*, *EZH2*	p		
	e-hChon-3	*ID2*, *ID3*, *E6*, *Bcl2*, *Core*, *Myc*, *Nanog*, *Sox2*	p		
	e-hChon-4	*Fos*, *Nanog*, *EZH2*	p	0/3	
Human dermal fibroblasts	e-hDFib1	*Myb*, *E7*, *HoxA9*, *Core*, *Myc*	p	0/3	
	e-hDFib2	*E7*, *MYC*	p		
	e-hDFib3	*ID2*, *FOS*, *ID3*, *E7*, *Core*, *ID1*, *MYC*	p		
Human foreskin fibroblasts	e-hFib-1	*RhoA*, *EZH2*	p		
	e-hFib-2	*Nanog*	p	0/3	
	e-hFib-3	*ID3*, *E7*, *Core*, *ID1*, *MYC*, *Yap1*, *Nanog*, *EZH2*, *Rex*	p		
Human lymphatic endothelial cells (dermis)	e-hLEC-1	*ID2*, *FOS*, *ID3*, *Core*, *Klf4*, *ID1*, *MYC*	p		
	e-hLEC-2	*MYC*, *ID1*, *ID2*	p		
Human microvascular endothelial cells (dermis)	e-hMEC-1	*ID2*, *Fos*, *TAg*, *ID3*, *E7*, *HoxA9*, *ID1*, *MYC*, *Nanog*, *Sox2*, *EZH2*, *Gli1*	p		
	e-hMEC-2	*ID2*, *FOS*, *ID3*, *BCL2*	p		
	e-hMEC-3	*ID2*, *FOS*, *ID3*, *HOXA9*, *ID1*, *Nanog*, *EZH2*	p		
Human osteoblast	e-hOB-1	*ID2*, *FOS*, *TAg*, *E7*, *MYC*	p		
	e-hOB-2	*Fos*, *TAg*, *Bcl2*, *MYC*, *Nanog*, *EZH2*, *Rex*	p	2/3	
	e-hOB-3	*ID2*, *TAg*, *E7*, *Myc*	p		
	e-hOB-4	*FOS*, *ßCAT*, *TAg*, *E7*, *ID1*, *MYC*	p		
Human bone marrow stroma cells	e-hStr-1	*ID2*, *FOS*, *ID3*, *E7*, *E6*, *Core*, *ID1*, *Lmo2*, *Yap1*, *Nanog*, *SOX2*, *EZH2*	p	0/3	
	e-hStr-2	*ID2*, *MYB*, *ID3*, *E7*, *E6*, *MYC*, *Yap1*, *Nanog*, *EZH2*	p		
	e-hStr-3	*ID2*, *Fos*, *E7*, *ID1*, *Lmo2*, *Yap1*, *Nanog*, *Sox2*	p	0/3	
	e-hStr-4	*ID2*, *E7*, *MYC*, *Nanog*, *SOX2*, *EZH2*	p		
	e-hStr-5	*E7*, *E6*, *Bmi1*, *ID1*, *MYC*, *Lmo2*, *Yap1*, *Nanog*, *SOX2*	p		
Human endothelial cells (umbilical cord)	e-hUVEC-1	*ID2*, *FOS*, *MYB*, *ID3*, *E7*, *ID1*, *MYC*, *Nanog*, *EZH2*, *Rex*	p		
	e-hUVEC-2	*MYC*, *ID1*, *ID2*	p	0/6	
	e-hUVEC-3	*Fos*, *Id3*, *Bmi1*, *Yap1*, *Nanog*	p		
	e-hUVEC-4	*SUZ12; ID4*, *Rex*	p		
	e-hUVEC-5	*ID2*, *FOS*, *ID3*, *E6*, *HoxA9*, *Core*, *Klf4*, *ID1*, *MYC*	p		
	e-hUVEC-6	*ID2*, *FOS*, *ID1*, *MYC*	p		
	e-hUVEC-7	*MYC*, *ID1*, *ID2*	p		
	e-hUVEC-8	*MYC*, *ID1*, *FOS*	p		
	e-hUVEC-9	*MYC*, *ID2*, *FOS*	p		
	e-hUVEC-10	*MYC*, *ID1*, *ID2*	p		
Murine hepatocytes	e-mHepA	*TAg*, *KLF4*, *ID3*, *Core*	c	0/3	0/11
	e-mHepB	*TAg*, *ID3*	p	3/3	3/3
	e-mHepC	*KLF4*	p	0/3	0/6

*exp* expansion, *p* polyclonal, *c* clonal, *s.c.* subcutaneous

Usually, a lag phase was observed at the beginning of the expansion period. Depending on the cell type, this state lasted between 20 and 40 days. Then, while the growth of mock-infected cells ceased, cells transduced with the gene library entered into a phase of continuous proliferation with doubling times ranging from 1.5 to 3.5 days. The cell lines reached 30 cumulative population doublings after 60–90 days (Fig. 1b). Typically, 10–40 proliferating clonal or polyclonal cell lines were obtained from 1 × 10^6^ primary cells. Of note, the cell lines showed no sign of senescence or crisis even during extended cultivation periods.

To investigate if cell expansion was accompanied with chromosomal rearrangements, we prepared consensus karyotypes from eleven cell lines. The human osteoblast cell line e-hOB-3 was examined both at early passage (passage 21) and after extended cultivation (passage 66). Ploidy changes were observed in four out of eleven tested cell lines (see Supplementary Fig. 1 for karyotype data and Supplementary Table 2 for a summary of results). No structural rearrangements were found in two out of eleven tested cell lines and while others showed rearrangement, only one was found to have more than three. Long-term cultivation of e-hOB-3 was accompanied by the gain of one additional structural change only, implying relative chromosome stability in vitro. Interestingly, structural rearrangements may have occurred non-randomly, targeting chromosome bands 2p16-24 and 22q13 in three out of eleven cell lines. Collectively, these analyses provided evidence that chromosomal evolution had not occurred during extended culture, but most likely alterations occurred and were selected during cell culture establishment. They thus can be considered as the most likely event underlying ploidy formation as observed among cancer cell lines^25^. To evaluate tumorigenicity we implanted seven cell lines subcutaneously into immunocompromised mice and monitored tumor formation. Apart from one osteoblast derived cell line, none of the other human cell lines gave rise to tumor formation within four months (Table 1).

The cell lines were evaluated for specific differentiation properties. Although pluripotency genes contributed to immortalization of some cell lines, none of the tested cell lines showed a pluripotent phenotype (Supplementary Fig. 2). Rather, the cells maintained differentiation specific properties as exemplified for four different donor derived cell types—osteoblasts, bone marrow stromal cells, microvascular endothelial cells, and chondrocytes (Supplementary Fig. 3).

To evaluate if specific genes or gene combinations facilitated cell expansion, we analyzed the gene integration profile of 29 human cell lines of various differentiation states including endothelial cells of umbilical cord and skin, chondrocytes, osteoblasts, fibroblasts, and bone marrow stromal cells. This analysis showed that on average 6–7 transgenes were integrated in the cell lines (Supplementary Fig. 4a). A set of eight genes (*ID1*, *ID2*, *ID3*, *MYC*, *FOS*, *E7*, *NANOG*, and *EZH2*) was integrated in more than 50% of these cell lines (Supplementary Fig. 4b), while others were rarely found. This suggested that a reduced subset of genes is sufficient for cell expansion.

### Immortalized hepatocytes maintain typical features

We further asked if defined cell types are preferentially immortalized with specific subsets of genes. To challenge this hypothesis, we used the full library and established 90 murine hepatocyte cell lines from three different genetic backgrounds and as control 15 fibroblastoid reticular cell (FRC) lines. The cell lines were analyzed for stably integrated genes from the library (Supplementary Figure 5). PCR analysis revealed an average of four genes per cell line for both, the hepatocyte and FRC lines. Fifteen genes out of the 33-gene library were not found in any of the cell lines. Interestingly, a cell type-specific pattern was observed for the 18 integrated genes: in the hepatocyte cell lines, the most frequently found genes comprise *KLF4* (88%), as well as *FOS*, *TAg*, *MYC,* and *ID3* (48–54%, Table 2). In the FRC lines the most frequently found genes were *FOS* (100%), *Core* (92%), *ID3* (58%), *REX1* (44%), and *TAg* (44%, Table 2). TAg contributed to the immortalization of both cell types with similar frequencies, whereas the other genes were found differentially integrated in hepatocytes and FRCs. This demonstrates that certain gene combinations preferentially expand these cell types. Of note, *MYC* and *KLF4* were not found in any of the FRC lines (Table 2), suggesting that some genes do not support or might even prevent cell expansion of a respective cell type.

**Table 2:** Integration frequency of genes into hepatocyte clones and fibroblastoid reticular cell (FRC) clones

Genes^*^	Integration frequency (%)	
	Hepatocyte clones (*n* = 90)	FRC clones (*n* = 15)
*KLF4*	88	0
*FOS*	54	100
*TAg*	52	44
*MYC*	48	0
*ID3*	48	58
*MYB*	34	17
*REX1*	33	44
*Core*	33	92
*SOX2*	25	8
*E7*	25	8
*EZH2*	19	6
*Yap1*	16	0
*Bmi1*	12	0
*Bcl2*	10	25
*HOXA9*	8	0
*GLI1*	3	6
*SUZ-12*	3	11
*ID1*	1	0

^*^genes that were not found in any of the cell lines are not included integration frequencies of >40% are highlighted

**Fig. 2:**
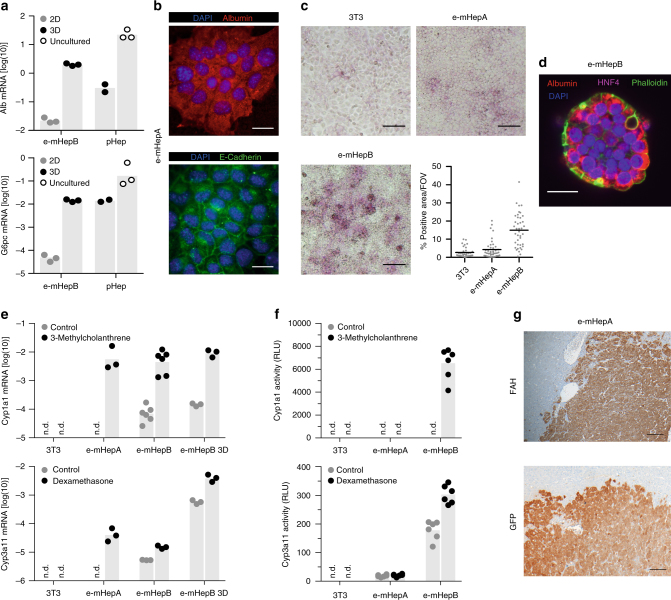
Hepatocyte cell lines retain key hepatic properties. **a** Albumin and Glucose-6-phosphatase gene expression analysis of expandable hepatocytes cultured in standard 2D or 3D (9 days of cultivation as spheroids in spinner flasks) conditions and primary hepatocytes (pHep) either cultured in 3D conditions or freshly isolated (expression normalized to Gapdh, *n* = 3 independent experiments, except for pHep were *n* = 2 independent experiments, bars represent mean). **b** Immunofluorescence-based detection of Albumin and E-cadherin in e-mHepA expandable hepatocytes counterstained for nuclei with DAPI. Scale bar, 20 μm. **c** Glycogen storage in expandable hepatocytes and control fibroblasts as analyzed by Periodic Acid-Schiff staining. Representative bright field micrographs and quantification of multiple field of views are shown. Scale bar, 100 μm (mean, pooled data from *n* = 3 independent experiments, field of views analyzed in total 35 to 42). **d** Immunofluorescence micrograph of Albumin and Hnf4α in e-mHepB hepatocytes after 3D culture as spheroids for 9 days. Actin filaments and nuclei were counterstained with phalloidin and DAPI, respectively. Scale bar, 20 µm. **e** Induction of phase I metabolic enzyme expression in expandable hepatocytes cultured in 2D or 3D conditions and control fibroblasts in response to stimulation with 50 μM Dexamethasone or 2 μM 3-Methylcholanthrene for 72 h (expression normalized to Gapdh, *n* = 3 independent experiments, except for e-mHepB Cyp1a1 expression were *n* = 6 independent experiments, bars represent mean). **f** Luminescence-based detection of Phase I enzymatic activity in expandable hepatocytes and control fibroblasts after cells were treated as indicate above (activity normalized to 10^4^ cells, pooled data from *n* = 2 independent experiments with *n* = 3 technical replicates each). **g** Staining of livers of *Fah*^–/–^/*Rag2*^–/–^*/Il2rg*^–/–^ (FRG) mice for FAH and GFP, 90 days after transplantation of GFP-tagged expandable hepatocytes. Scale bar, 100 µm

To investigate if the immortalization procedure allows the preserving of typical cell properties, we characterized the established hepatic cell lines for key hepatic markers and functions. The prototypic cell lines e-mHepA (*KLF4*, *TAg*, *ID3*, *Core*) and e-mHepB (*TAg*, *ID3*), were chosen for further characterization. Both lines showed epithelial cobblestone-like morphology and albumin mRNA expression. These cell lines also expressed the mature liver marker glucose-6-phosphatase (G6pc), as well as other key hepatic markers such as Hnf4a, Krt18, and Cebpa (Fig. 2a,b and Supplementary Fig. 6a). The expanded hepatocytes also expressed the lateral surface marker E-cadherin that indicates epithelial polarization (Fig. 2b). Both hepatic cell lines stored glycogen demonstrated by patches of periodic acid Schiff staining-positive cells (Fig. 2c).

Like primary hepatocytes, the immortalized hepatocytes maintained the capacity to form three-dimensional (3D) cell aggregates (spheroids) of defined size upon cultivation in spinner flasks (Supplementary Fig. 6b). Of note, culturing cells using 3D conditions increased expression of Albumin and G6pc by 88-fold and 290-fold, respectively, to levels comparable to primary murine hepatocytes cultured in 3D conditions (Fig. 2a). Immunostaining of spheroids also confirmed expression of Albumin and Hnf4α proteins with F-actin being localized to intercellular borders (Fig. 2d). Global gene expression profiling demonstrated upregulation of hepatic markers when cells were cultured in 3D conditions relative to standard 2D cultivation, though most hepatic markers were expressed at lower levels compared to freshly isolated primary hepatocytes (Supplementary Fig. 7). Interestingly, the increase of hepatic marker expression in 3D was accompanied by a reduction of proliferation as shown by decreased Ki67 expression (Supplementary Fig. 6c). This is in line with the pronounced hepatic phenotype of non-replicating hepatocytes in vivo^26^.

To test phase I metabolic capacity of the expandable hepatocytes and their ability to modulate cytochrome P450 monooxygenase (Cyp450) expression via nuclear receptor signaling, expression and activity of Cyp3a11 (murine homolog of CYP3A4) and Cyp1a1 was analyzed (Fig. 2e,f). As exemplified for e-mHepA and e-mHepB cells, transcription of Cyp1a1 and Cyp3a11 was upregulated upon stimulation with the aryl hydrocarbon receptor (AhR) agonist 3-Methylcholanthrene or the pregnane X receptor (PXR) agonist dexamethasone, respectively. Interestingly, the expression of Cyp3a11 in e-mHepB cells was further increased 100-fold when they were cultured in 3D conditions, whereas expression of Cyp1a1 remained constant. Upon stimulation with their respective inducers, Cyp1a1 enzymatic activity was strongly upregulated in e-mHepB cells, while Cyp3a11 activity was slightly increased (Fig. 2f), suggesting functional nuclear receptor signaling.

Despite unlimited in vitro expansion potential of the three hepatocytic cell lines (e-mHepA, e-mHepB, and e-mHepC, with the genes *TAg/KLF4/ID3/Core*, *TAg/ID3*, and *KLF4*, respectively), only e-mHepB showed tumorigenicity as assessed by subcutaneous injection into immunodeficient mice (Table 1).

To examine the ability of the expanded hepatocytes to engraft into a damaged liver, the three hepatic cell lines e-mHepA (eGFP-tagged), e-mHepB, and e-mHepC were transplanted intrasplenically into immunodeficient *Fah*^–/–^*/Rag2*^–/–^*/Il2rg*^*–/–*^ (FRG) mice^27^. Islets of Fah-positive (and eGFP-positive for e-mHepA) cells were observed for all cell lines 12 weeks after transplantation, similar to control primary murine hepatocytes (Fig. 2g and Supplementary Fig. 6 d,e,f), suggesting successful engraftment. Furthermore, the expandable cell lines retained their proliferation potential in vivo as indicated by Ki67-positive-stained islets of engrafted cells (Supplementary Fig. 6f). While expandable hepatocytes successfully engrafted into the liver parenchyma, survival of FRG mice was not improved. Taken together, the expansion of hepatocytes was compatible with the prolonged expression of key functions and the ability to integrate into the liver parenchyma.

### Cell expansion of endothelial cell lines with gene subsets

**Fig. 3:**
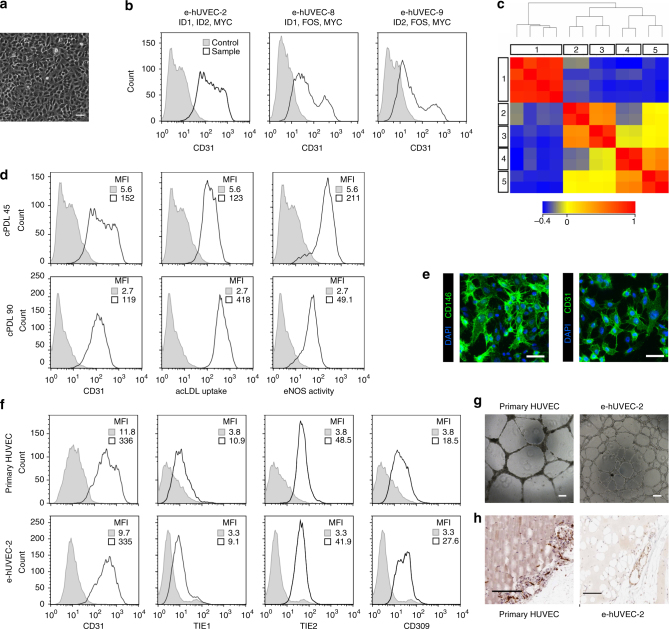
Comparable phenotype of primary and expanded endothelial cells. **a** Phase contrast microscopy shows the expandable HUVEC cell line e-hUVEC-2 (MYC, ID1, and ID2). Scale bar 100 µm. **b** CD31 expression of human endothelial cell populations immortalized with three different gene sets as indicated. **c** Global gene expression analysis was performed on three different HUVEC lines (e-hUVEC-2, 8 and 9; in duplicate) (3, 4, 5, respectively) and two independent primary HUVEC populations (2) as well as four primary gingiva fibroblast populations (1). Expression data was processed with GeneSpring 11.5.1 software and a Standard Pearson Correlation was determined for each gene versus all other genes. The correlation heatmap depicts the pair-wise correlation coefficient between the given samples and displays the relationship between the different samples. The samples are clustered based on the pair-wise correlation coefficients between all entities. **d** Phenotypic stability of e-hUVEC-2 cells after 45 and 90 cumulative population doublings was evaluated by CD31 (also known as PECAM1) expression, acetylated LDL uptake, and eNOS activity. Gray fill: antibody isotype control; black outline: stained sample. MFI: median fluorescence intensity. **e** Immunofluorescence-based detection of CD31 and CD146 (also known as MCAM) in expandable HUVECs counterstained for nuclei with DAPI. Scale bars, 100 µm. **f** The phenotype and the functionality of cell line e-hUVEC-2 (cumulative population doubling 80) was compared to primary HUVECs based on flow cytometric analysis of CD31, TIE1, TIE2, and CD309 (also known as VEGFR2) expression. Gray fill: antibody isotype control; black outline: stained sample. MFI: median fluorescence intensity. **g** The angiogenic potential of primary HUVECs and e-hUVEC-2 was determined in vitro by a matrigel tube formation assay. Scale bars, 200 µm. **h** Spheroids in matrigel of primary HUVEC and e-hUVEC-2 were subcutaneously injected into *Rag2*^-/-^*Il2r*g^−/−^ mice. After two weeks the implants were dissected and stained for human CD31 (brown color). e-hUVEC-2 organized into human CD31 positive microvessels similar to primary HUVEC. Scale bars, 100 µm

The profiles of integrated genes as described above (see Table 1 and Supplementary Fig. 4) suggest that the immortalization of different cell types requires specific combinations of genes. Therefore, we asked whether this approach allows identification of a minimal gene combination that is sufficient for robust and reproducible immortalization of a certain cell type. For this purpose, we generated expandable human umbilical vein endothelial cells (HUVECs) with the whole gene library and identified nine genes in the resulting cell line (e-hUVEC-1): *ID2*, *FOS*, *MYB*, *ID3*, *E7*, *ID1*, *MYC*, *NANOG*, and *REX*. Next, primary HUVEC cells were infected with randomly chosen subsets from these nine genes. From two out of nine different gene combinations, we could establish immortalized cell lines expressing the endothelial marker protein CD31 (eHUVEC-5 and eHUVEC-6). Both cell lines contained four of the transduced genes in common, namely *ID2*, *FOS*, *MYC*, and *ID1*. Therefore, subsequent infections of primary HUVECs were performed with different subsets of these four genes. Three combinations (*MYC/ID1/ID2*, *MYC/ID1/FOS*, or *MYC/ID2/FOS*) gave rise to CD31-positive cell lines. We observed that the cell lines immortalized with *MYC/ID1/FOS* (e-hUVEC-8) or *MYC/ID2/FOS* (e-hUVEC-9) displayed pronounced cell-to-cell heterogeneity with respect to CD31 expression. In contrast, endothelial cell lines immortalized with the gene set *MYC/ID1/ID2* (e-hUVEC-2) showing typical cobblestone morphology (Fig. 3a) were found to express the marker CD31 homogenously (Fig. 3b).

The phenotypic similarities between expanded and primary HUVECs were analyzed by global gene expression profiling. The analysis identified a positive correlation between the HUVEC lines (e-hUVEC-2; 8; 9) with the primary HUVECs and a negative correlation with control fibroblasts. Additionally, the HUVEC cell lines clustered with the primary HUVECs (Fig. 3c). This was also confirmed by the principal component analysis that revealed a high similarity between expanded and primary HUVECs in the first principal component (Supplementary Fig. 8). Further data analysis confirmed the endothelial phenotype by a pronounced expression of endothelial specific marker genes (Supplementary Fig. 9).

Due to their homogeneous CD31 expression, e-hUVEC-2 cells were selected for in depth characterization of endothelial properties. When we analyzed e-hUVEC-2 cells for nitric oxide (NO) production and acetylated low-density lipoprotein (acLDL) uptake, we found comparable levels after 45 and 90 cumulative population doublings demonstrating a stable phenotype upon cultivation (Fig. 3d). Furthermore, expression of CD31 and CD146 was confirmed by immunofluorescence (Fig. 3e). Of note, the cells induced inflammation dependent marker proteins (ICAM1, E-Selectin, and VCAM) upon stimulation with TNFα (Supplementary Fig. 10). Interestingly, the HUVEC line showed a dose dependent increase in proliferation when cultivated with VEGF and bFGF, factors that are known to induce endothelial cell proliferation, and could be blocked by the respective inhibitor (Supplementary Fig. 11). In addition, we compared the e-hUVEC-2 cells side-by-side to primary HUVEC with respect to the expression of endothelial markers CD31, TIE1, TIE2, and CD309. Notably, the expanded cells expressed the markers to a similar degree as the primary cells (Fig. 3f).

We next analyzed the ability of the novel cell systems to form vessel like structures. In vitro, the cells could form tube-like structures when embedded in Matrigel, comparable to those formed by primary cells (Fig. 3g). In addition, a recently described transplantation assay^19,28^ was employed to analyze whether the HUVEC cell lines would fulfill their function in vivo. Using this setting we obtained vessels which stained positive for human CD31, demonstrating the functional capability of the expanded HUVEC in vivo (Fig. 3h). Of note, no tumor formation was observed upon further monitoring of the mice for 6 months (Table 1).

### Reproducibility of immortalization and cellular phenotypes

**Fig. 4:**
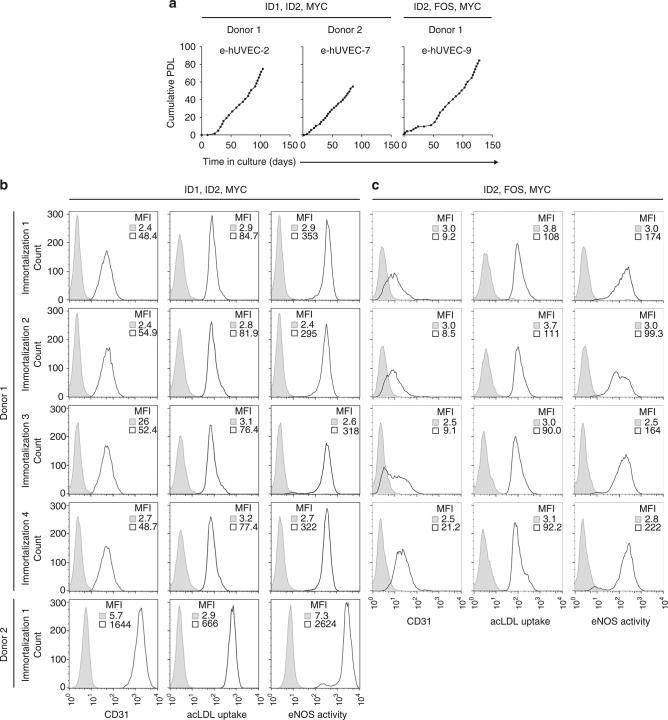
Cell-type-specific and reproducible cell expansion. **a** Cumulative population doubling levels of HUVEC lines e-hUVEC-2, e-hUVEC-7, and e-hUVEC-9. For e-hUVEC-2 and e-hUVEC-7, primary cells were derived from different donors, but generated using the same gene set (ID1, ID2, and MYC). e-hUVEC-9 cells were transduced with ID2, FOS, and MYC. Graphs for e-hUVEC-2 were taken from Fig. 1b. **b** Four independent endothelial cell lines generated from the same donor upon transduction with MYC, ID1, and ID2 (technical replicates to e-hUVEC-2), and one cell line generated using the same gene set but derived from a different donor (biological replicate to e-hUVEC-2), were analyzed after 80 cumulative population doublings for acLDL uptake, CD31 expression, and eNOS activity. **c** Four independent endothelial cell lines generated from the same donor upon transduction with ID2, FOS, and MYC (technical replicates to e-hUVEC-9) were analyzed after 80 cumulative population doublings for acLDL uptake, CD31 expression, and eNOS activity. Unstained or isotype control is shown in gray, antibody stained samples are depicted in black

To validate the reproducibility of immortalization with a given set of genes with respect to the properties of the generated cell lines we established four technical replicates by immortalizing primary human umbilical cord cells of a given donor with the same set of genes (MYC, ID1, and ID2). To assess the reproducibility among various donors, biological replicates were generated by employing the same set of genes to immortalize cells from a second donor. These cells were also readily immortalized. As a control, we used the independent gene set *MYC*, *ID2*, and *FOS*, which displayed a heterogeneous CD31 expression pattern, comparable to the cell line e-hUVEC-9, which was established by the same gene set. In all cases, robustly proliferating cell lines were established with this gene combination (Fig. 4a for representative growth curves). In addition, we achieved high reproducibility of marker expression in the technical replicates with both gene combinations (Fig. 4b, c). Importantly, functional analysis revealed that all of the analyzed cell lines from technical as well as from the biological replicates displayed a comparable phenotype even after prolonged cultivation (80 cumulative populations). Together, these data show paradigmatically for endothelial cells the reproducibility in cell line establishment with regard to immortalization and cell phenotype.

## Discussion

Highly reproducible and expandable cell cultures that can be rapidly derived from tissues of individuals are an essential tool for research and drug development, in particular for personalized medicine. Current sources of personalized cell systems concern primary cells and iPS cells derived from individual donors. The low proliferation capacity of primary cultures and differentiated cells derived from embryonic stem cells and iPS cells is an obvious drawback and limits their routine use.

In our study, we used a lentiviral library consisting of 33 genes for the generation of long-term proliferating cell lines. Applying a simple screening protocol, we derived cell lines from all the tested mouse and human derived tissues. For every investigated cell type, cell lines could be generated that show authentic, i.e., functional, phenotypes. This demonstrates that this technology is widely, if not universally, applicable and supports functional cell expansion. This result could be reproduced with defined subsets of these genes indicating that different cell types require different subsets. This is supported by distinct sets of genes that were found in murine hepatocytes versus the FRCs. As exemplified for endothelial cell lines, an appropriate subset of genes can lead to reproducible cell line generation while preserving the primary phenotype. 3D culture and grafting experiments (shown here for the hepatocyte cell lines) further demonstrated that cell type specific properties, which were almost lost during conventional 2D in vitro cultivation, can be restored if adequate culture environments are provided for the cells. The grafting capacity as demonstrated for hepatocytes and endothelial lines confirmed the flexibility and authenticity of these cells and argues in favor of intact cell–cell interactions and physiological reactiveness.

Although complete reproducibility for this immortalization cannot be expected due to variable transgene expression, based on position effects upon random gene integration and variable copy numbers^29,30^, we observed high reproducibility of the cell line generation process giving rise to cell lines with similar phenotypical properties. The underlying mechanism of the reproducible cell line generation process is not well understood. We assume that it is attributed to the selection process applied, which favors the proliferation of cell lines with adequate expression levels of the relevant genes. The contribution of the individual genes to conferring properties that lead to immortalization is difficult to assess. While certain genes are known to push cells into proliferation, others like the pluripotency genes, might indirectly contribute by moving cells toward their proliferating precursors. The gene library contains well-known immortalizing genes like *TAg* and *E7*. While the creation of many cell types was possible without these genes (hepatocytes, endothelial cells, and fibroblasts from different sources, as well as chondrocytes) all isolated BM stroma cells and osteoblasts contained *E7* and *TAg*, respectively. This suggests that expression of these genes is required for immortalization. However, it is unlikely that they act alone, since we never found cell clones exclusively containing these genes. We assume that they act in concert with the other immortalizing genes. This might also be the reason why none of the cell lines shows a transformed phenotype and only one line was found to form tumors in immunocompromised mice.

The new technology as presented here allows the reproducible cell expansion of primary cells, overcoming the unpredictability that is typically associated with previous cell line development procedures. It enables the generation of personalized cell lines from only 1×10^6^ primary cells within 60–90 days. These cultures are clonable, easily expandable, and hence available for multiple applications. For example, the HUVEC derived cell lines generated in this study provide a stable phenotype during prolonged cultivation periods of >90 population doublings. Thereby, theoretically 1×10^27^ cells can be generated from a single experiment. This is far beyond the needs of industrial screenings and other drug development processes, which usually require between 10^10^ and 10^11^ cells.

Since DNA is most vulnerable to errors in the S phase, extended proliferation per se is accompanied by the acquisition of mutations and chromosomal aberrations. This applies to all types of cells including embryonic stem cells and iPS cells in culture as shown by genetic profiling, underscoring the need for “younger” cell lines^31,32^. The robustness, simplicity, and reproducibility of the presented cell line generation technology enables the reiterated establishment of low passage cell lines with predictable properties. In this regard, the method for cell expansion also overcomes the problem of chromosomal instability even for cell types in which primary cell expansion is associated to the loss of phenotypic properties.

Together, the reproducibility and the short timelines of the novel cell expansion approach enable the creation of test systems for many applications including drug discovery, toxicity testing, as well as for disease understanding and personalized medicine.

## Methods

### Derivation of primary cells

The following primary cell types were used for the cell expansion procedure:

Human adult dermal fibroblasts, HUVEC, human dermal microvascular endothelial cells (HMVEC), and human lymphatic endothelial cells (LEC) were purchased from Promocell GmbH (Heidelberg, Germany) and adult human bone marrow cells were purchased from Lonza GmbH (Cologne, Germany).

The Hepa 1–6 (ACC-175) and NiH 3T3 (ACC-59) cell line were obtained from DSMZ. Primary human foreskin fibroblasts (FS4 cells)^33^ were a kind gift by Rentschler GmbH.

Adult human bone marrow cells (hBMSCs) were isolated from bone marrow biopsy cells and purified using gravity sedimentation in 0,1% methylcellulose followed by erythrocyte lysis with ammonium chloride^34^. The nucleated cell fraction was subsequently seeded and used for transduction experiments. Primary human chondrocytes were obtained from human cartilage from the femoral head. The cartilage was fractionated and incubated with pronase (0.28 U/ml, Roche) and hyaluronidase (0.108 U/ml, Sigma) solution for 1 h at 37 °C under agitating. After washing in PBS, the cartilage was digested by collagenase (25 U/ml, Sigma) for 1 h at 37 °C under agitating. Debris was eliminated by a cell strainer (BD). The isolated cells were washed twice with PBS and cultivated in Ham’s F12 (PAA) supplemented with 10% FCS (PAA), 1% penicillin/streptomycin (PAA), 1% amphotericin B (Sigma) and 50 mM ascorbic acid (Sigma). Human trabecular bone cylinders of the central part of the head were obtained with a trephine, cut in small samples, and washed extensively in PBS. The primary explant technique was used to obtain osteoblast cultures from hip and knee samples^35^.

Murine lymph node fibroblastic reticular cells (FRCs) from skin-draining/peripheral (inguinal and axillary) or mesenteric lymph nodes^36^ were isolated from BALB/c mice (Janvier). Lymph nodes of 10 mice were digested in 5 ml 1640-RPMI (Gibco) containing 0.2 mg/ml collagenase P (Roche), 0.15 U/ml dispase (Roche) and 0.2 mg/ml DNase I (Roche). After digestion, the cells were kept in 4 °C PBS containing 0.2 % w/v albumin bovine serum (Sigma) and 5 mM EDTA (Roth). The cells were enriched for CD45^−^ negative cells via labeling of hematopoietic cells with CD45-APC (clone: 30-F11, eBioscience) and subsequently labeled with anti-APC microbeads both for 20 min at 4 °C with MACSmix^TM^ (Miltenyi). CD45^+^ depletion was achieved utilizing autoMACS^®^ (Miltenyi). The CD45-depleted cells were sorted for CD45^−^CD24^−^CD31^−^gp38^+^ FRCs via FACS AriaII (100-μm nozzle). 2×10^4^ FRCs were plated on 24-well plates (Thermo Scientific Nunc) coated for 2 h at 37 °C with 0.05 mg/ml fibronectin (Roche).

Murine hepatocytes were isolated from various mouse strains by using a two step perfusion protocol^37^ (reviewed in^38^). In brief, the mice were narcotized with ketamine/xylazine (0.1 ml per 10 g body weight). The catheter was fixed in the portal vein and connected to the external pump system. Liver perfusion was processed by using 125 ml prewarmed (37 °C) liver perfusion medium (Gibco) supplemented with heparin (2500 U/ml) with a flow rate of 8 ml/min. The vena cava inferior was opened to enable the emission of the medium. Enzymatic digestion was performed by application of 125 ml liver digest medium (Gibco) supplemented with 5 g Liberase^™^ (Roche) with a flow rate of 25 ml/min. Upon complete digestion of the liver, the liver was resected and placed in a petri dish filled with precooled (4 °C) Dulbecco’s Eagle Medium (DMEM) (Gibco) supplemented with 10% fetal bovine serum (FBS) (Lonza), 100 U penicillin (Gibco), 100 µg/ml streptomycin (Gibco), and 2 mM glutamine (Gibco). The liver lobes were carved and the hepatocytes were gently shaken out of the liver. The suspension was passed through a 100 µm cell strainer to remove liver tissue. Hepatocytes were purified and washed by centrifugation at 50x* g*, 4 °C, for 5 min without break. The supernatant (non-parenchymal fraction) was discarded and the hepatocyte pellet was resuspended in the appropriate medium for cultivation and counted by the GUAVA EASYCyte^TM^.

For the isolation and expansion of human primary cells (bone marrow stroma cells, chondrocytes, and osteoblasts) tissues were used with ethical approval by the respective local institutional review board (RWTH Aachen: EK135/09; University Hospital Essen; Hospital U.M. Valdecilla) and patients’ informed consent. Isolation of mouse hepatocytes by in situ perfusion was approved by the Niedersächsiches Landesamt für Verbraucherschutz und Lebensmittelsicherheit.

### Cell culture

Primary cells and cell lines were maintained at 37 °C in a humidified atmosphere with 5% CO_2_ and regularly screened for the absence of mycoplasma contamination. The following media were used for cultivation of fibroblasts: huFib medium (InSCREENeX GmbH), bone marrow stroma cells: huMSC medium (InSCREENeX GmbH), HUVECs: huVEC medium (InSCREENeX GmbH), HMVECs: huMEC medium (InSCREENeX GmbH), LECs: huLEC medium (InSCREENeX GmbH), chondrocytes: huChon medium (InSCREENeX GmbH), and osteoblasts: huOB maintenance medium (InSCREENeX GmbH).

Murine lymph node FRCs were cultivated in RPMI (Gibco) supplemented with 10% fetal calf serum (FCS) (Biochrom), 100 U penicillin (Gibco), and 100 µg/ml streptomycin. For cultivation of murine hepatocytes Dulbecco’s Eagle Medium (DMEM) (Gibco) supplemented with 10% FBS (Lonza), 100 U penicillin (Gibco), 100 µg/ml streptomycin (Gibco), and 2 mM glutamine (Gibco) or hepatocyte cultivation medium (HCM) (Lonza Ltd., Switzerland) supplemented with 2% FBS (Lonza Ltd., Switzerland), respectively, was used.

Endothelial cells were cultivated in gelatin-coated tissue-culture flasks. For this purpose the cell culture plates were covered with a 0.5% gelatin solution (InSCREENeX GmbH) for 30 minutes at 37 °C. The gelatin solution was aspirated and the endothelial cells were plated. Murine FRCs were cultivated in fibronectin-coated tissue-culture dishes. For this purpose, the culture dish was covered with fibronectin (Roche) solved in 2 M urea (1 mg/ml) for 2 h at 37 °C. The murine hepatocytes were cultivated in cell culture dishes that were coated with 0.2% collagen solution (InSCREENeX GmbH).

3D cultivation of chondrocytes was performed in hanging drop cultures. For this purpose, 2×10^4^ cells (50 µl drop) from a single cell suspension were plated in the lid of a tissue culture plate that was carefully turned and cultivated for five days with media exchange after two days.

3D cultivation of hepatocytes was performed in spinner flasks. 2×10^7^ cells/100 ml medium were used for inoculation. The cultures were agitated with 10x *g* and received regular media exchange. To determine the size, the spheroids were visualized by phase contrast microscopy. The average diameter was determined by measuring three diameters per spheroid with ImageJ software. The average diameter was calculated by the following equation: $d_{\mathrm{average}} = \left( {d_1 \times d_2 \times d_3} \right)^{\frac{1}{3}}$.

### Lentiviral vectors

The cDNAs from the genes listed in Supplementary Table 1 were obtained from Biocat GmbH (Heidelberg; Germany) and implemented into a third generation self-inactivating lentiviral vector^39^. Expression of the genes is controlled by an internal SV40 promoter. Some lentiviral vectors additionally encode a neomycin resistance gene expressed from a bicistronic message using the Poliovirus internal ribosomal entry site element. This concerns vectors encoding *MYC; CTNNB1; ID1; TAg; Fos; E2F1; JUN; NS1; SOX2; ID2; Lmo2; Nanog; NFE2l2; Yap1; E7; E6; GLI1; SUZ12; EZH2; ZNF217; RHOA; MYCN; ID4*. Integrity of all cDNAs was confirmed by DNA sequencing.

Virus production was performed for each lentiviral vector separately by transient calcium phosphate transfection of HEK293T cells using four plasmids encoding the helper functions (gagpol, rev, VSV-G) and the respective lentiviral vector^40^. The titre evaluated on NIH3T3 cells ranged between 1×10^6^–5×10^6^ infectious particles per ml for the different viruses. For immortalization experiments, mixtures of all or subsets of library genes were generated and stored at −70 °C until use.

### Cell expansion

For cell expansion, the primary cells were seeded one day prior to infection on 48-, 12- or 6-well plates and infection was done with an MOI between 1 and 5. The cell number of the primary cells was adjusted so that the cells reached 70–80% confluence at the day of infection. For the infections lentiviral vector preparations were mixed with culture medium of the respective primary cell. The culture media made up at least 50%. All infections were performed in the presence of polybrene in a final concentration of 8 µg/ml. After 8–12 h, the virus was removed and cells were further cultured. For all infections, a mock-infected control was included to determine the time point at which the primary cells enter crisis. For routine culture of the cell lines, the cells were passaged when they reached 80–90% confluence. Polyclonal cell lines were obtained from pooled populations of proliferating cells. For establishment of individual hepatocyte and FRC cell clones, the primary cells were split 7–14 days after infection at low densities. Emerging cell clones were picked manually and clonal populations were expanded.

This cell expansion strategy provided the option to select for infected cells by applying G418, which eliminates all non-infected cells, or to select for the growth advantage of the transduced cells. Generally, we used G418 selection (0,2 mg/ml–0,4 mg/ml) for primary cells, which exhibit strong proliferation capacity (fibroblasts, HUVEC), while selection for growth advantage was used for those primary cells exhibiting lower proliferation capacity (osteoblasts, chondrocytes, bone marrow stroma cells, microvascular and lymphatic endothelial cells, and hepatocytes).

Cumulative population doublings were calculated according to the recommendation of the American Type Culture Collection (ATCC) based on *n* = 3.32 (log UCY–log l) + *X*, with *n* = the final PDL number at end of a given subculture, UCY = the cell yield at that point, *l *= the cell number used as inoculum to begin that subculture, and *X* = the doubling level of the inoculum used to initiate the subculture being quantified.

### Identification of the integrated transgenes

To identify the genes that were integrated into the newly established cell lines a PCR strategy was developed. For this purpose, 33 independent PCR reactions were performed using an universal 5′ primer located in the SV40 promoter and a unique 3′ primer specific for the respective gene (see Supplementary Table 2). Genomic DNA was isolated according to the following procedure. 1 × 10^5^–1 × 10^6^ cells were washed twice with PBS and incubated with 500 µl of lysis buffer (10 mM Tris, pH 7.5, 10 mM EDTA, 10 mM NaCl, 0.5% sodium dodecyl sulfate, and 1 mg/ml proteinase K). The cell lysate was transferred to a 1.5 ml plastic tube and incubated overnight at 60 °C. The next day, 1 ml 75 mM sodium acetate (in ethanol) was added, incubated at room temperature for 2 h. The DNA pellet was centrifuged in a table top centrifuge for 5 minutes at 2200x* g* and washed with 70% ethanol twice. After the final wash, the pellet was allowed to dry and dissolved in 30–50 µl TE (10 mM Tris, pH 7.5, 10 mM EDTA) and stored at 4 °C.

For PCR the Mango-Taq Polymerase Kit (Bioline GmbH, Germany) was used with the following conditions (15 µl final reaction volume: 7.5 µl Biomix (Bioline), 1.5 µl 5′ primer (10 µM), 1.5 µl 3′ primer (10 µM) 0.5 µl DMSO, 4.5 µl H_2_O, and 0.5 µl template DNA (100–1000 ng)): 95 °C (5 min), 72 °C (10 min), then 25 cycles at 95 °C (30 s)/55 °C (45 s) / 72 °C (240 s), and a final extension at 72 °C for 10 min. Non-infected cells were used as negative control. The PCR reactions were analyzed on a 1% agarose gel and scored for presence or absence of fragments of respective sizes to identify the integrated genes.

### Flow Cytometry

For the analysis of endothelial surface markers, the cells were disaggregated with trypsin/EDTA (0.05%, 0.02%) (Biochrom, Germany) and then incubated with fluorescently labeled monoclonal antibodies: CD31 (eBioscience; cat.no. 11–0319; dilution 1:100); CD309 (BD Pharmingen; cat.no. 560494; dilution 1:100); CD54 (eBioscience; cat.no. 12–0549; dilution 1:100); CD62e (eBioscience; 12–0627; dilution 1:100); CD106 (eBioscience; cat.no. 12–1069; dilution 1:100); as well as isotype control antibodies. The cells were incubated in 2% FBS (Biochrom, Germany) in PBS for 30 minutes at room temperature using concentrations according to the manufacturer’s instructions. Staining for Tie1 (Abcam; cat.no. ab 27851; dilution 1:100) and Tie2 (BD Pharmingen; cat.no. 557039; dilution 1:100) was performed by incubating the cells with the primary antibodies for 30 minutes at room temperature, washing with PBS and incubating with a phycoerythrin (PE)-labeled anti-mouse antibody (Dianova, Germany; cat.no. 715-116-151; dilution 1:200) for 30 minutes at room temperature. Afterwards, the cells were washed with 2% FBS in PBS and centrifuged. Finally, the cell pellet was resuspended in 2% FBS in PBS and analyzed by flow cytometry.

To assess the uptake of acLDL, the cells were cultivated for 4 h at 37 °C with 4 µg/ml acLDL (labeled with Bodipy FITC, Molecular Probes). After incubation, the cells were washed twice with PBS, disaggregated with trypsin/EDTA (0.05%, 0.02%) (Biochrom, Germany) and centrifuged. The cell pellet was resuspended and analyzed by flow cytometry. For the determination of the activity of the eNO synthase (eNOS) the cells were incubated for 30 minutes at room temperature with DAF-2 DA (1 µM, Enzo Life Sciences). Afterwards the cells were washed twice with PBS, disaggregated with trypsin/EDTA and centrifuged. The cell pellet was resuspended and analyzed by flow cytometry. Cell debris was excluded by gating for FSC > 200 in SSC/FSC. The gated cells were analyzed for the respective fluorescence signals and plotted as histograms.

### In vitro angiogenesis

In vitro angiogenesis was monitored by a standard matrigel assay^18^. Briefly, 96-well plates were coated with 100 µl matrigel (BD Biosciences) for 30 min at 37 °C. 4×10^4^ cells were seeded per well and cultivated overnight. Tube structure formation was evaluated by microscopic analysis.

### Immunofluorescence and microscopy

For immunofluorescence 3D hepatocyte spheroids were harvested by centrifugation at 50x *g* for 5 min without break. The cells were fixed with ice-cold methanol/acetone (1:1) solution for 5 min. Primary antibodies (anti-mouse albumin; abcam cat.no. ab135575; dilution 1:100; anti-mouse HNF4α ThermoFisher cat.no. MA1-199; dilution 1:1000) as well as phalloidin (Alexa488 conjugated, Molecular Probes cat.no. A12379; dilution 1:100) were diluted in 0.1% saponin and incubated on the washed cells for 60 min at room temperature. The secondary antibodies (goat anti-rabbit Cy3; Dianova cat. no. 111-166-045; dilution 1:100; goat anti-mouse Cy5 from Dianova cat.no. 115-175-166; dilution 1:100) were diluted in 0.1% saponin and incubated with the cells for 60 min at room temperature. Stained cells were embedded in fluoroshield (Sigma) containing DAPI. Upon incubation and drying overnight at 4 °C in the dark, the stained cells were examined using the confocal microscope LSM 510 Meta (Zeiss).

For determination of glycogen storage, the cells were fixed with 4% paraformaldehyde for 20 min and afterwards, washed 3 times with water. Periodic acid (10 ng/ml) (Sigma) was used for 5 min for oxidization followed by the treatment with Schiff’s reagent (Sigma) for 15 min to visualize the aldehyde groups.

### Gene expression analysis

For expression analysis, total RNA was extracted from harvested cell pellets using the RNeasy Mini Kit (Qiagen) in combination with QIAschredder (Qiagen). 5 µg of total RNA were reversely transcribed using the Ready-To-Go First Strand Beads (GE Healthcare) and OligodT primers.

qRT-PCR was performed with Qiagen SYBR Green RT-PCR Mix on Light Cycler 480 (Roche). Murine gene-specific primers are specified in Supplementary Table 4. Expression levels were normalized to the mRNA of the housekeeping gene GAPDH.

DNA microarray hybridization and analysis was performed at HZI array facility. Prior analysis, quality and integrity of total RNA was controlled on Agilent Technologies 2100 Bioanalyzer (Agilent Technologies; Waldbronn, Germany). 500 ng of total RNA were used for biotin labeling according the 3′ IVT Express Kit (Affymetrix; Santa Clara, CA). 7.5 µg of biotinylated cRNA were fragmented and placed in a hybridization cocktail containing four biotinylated hybridization controls (BioB, BioC, BioD, and Cre) as recommended by the manufacturer. Samples were hybridized to an identical lot of Affymetrix GeneChip HG-U133 Plus 2.0 for 16 h at 45 °C.

Steps for washing and SA-PE staining were processed on the fluidics station 450 using the recommended FS450 protocol (Affymetrix; Santa Clara, CA). Image Analysis was performed on GCS3000 Scanner and GCOS1.2 Software Suite (Affymetrix; Santa Clara, CA).

Alternatively, 50 ng of total RNA were applied for Cy3-labeling reaction using the one color Quick Amp Labeling protocol (Agilent Technologies; Waldbronn, Germany). Labeled cRNA was hybridized to Agilent´s murine 4 × 44k V2 microarrays for 16 h at 68 °C and scanned using the Agilent DNA Microarray Scanner.

Analysis of microarray data was performed using GeneSpring 11.5.1 (Agilent Technologies, CA, USA). Signal intensities (raw data) were log2 transformed and normalized using RMA. Gene expression values per condition are given as relative expression to mean expression value calculated from intensities of all conditions (mean centralization, normalized data).

To assess the endothelial phenotype of endothelial cell lines the gene arrays were analyzed based on a set of endothelial genes^41^.

### Cytochrome P450 activity

Activity of Cytochrome P450 enzymes was analyzed using the P450-Glo CYP1A1 (Luciferin-CEE) and CYP3A4 (Luciferin-PFBE) assay kits (Promega) according to manufacturer’s instructions (cell-based, lytic protocol). Briefly, the cells were treated with vehicle control, 50 µM Dexamethasone or 2 µM 3-Methylcholanthrene for 72 h with daily media changes before medium was replaced to contain the respective CYP luminogenic substrates. The cells were incubated for 3 h before detection reagent was added and luminescence was measured using a Lumat LB 9507 luminometer (Berthold Technologies) and an integration time of 10 s. The luminescence signals were background corrected (medium without cells) and normalized to cell numbers.

### Animals and cell transplantation experiments

Animal experiments were performed in accordance to the German animal welfare law and permission by the Niedersächsiches Landesamt für Verbraucherschutz und Lebensmittelsicherheit. Male and female mice used for experiments in this study were aged between 8 to 12 weeks, unless stated otherwise.

Mouse strains RosaConL^42^, C57BL/6 (Jackson Laboratory), BALB/c mice (Janvier) were used for primary hepatocyte isolation. Immunodeficient, fumaryl hydrolase-deficient Fah^−/−^/Rag2^−/−^/Il2rg^−/−^ mice (FRG)^27^ were used as recipients for hepatocyte cell transplantation. Mice were maintained with NTBC (Swedish Orphan Biovitrum GmbH) in the drinking water prior to the experiments. Transplantation was performed using the collagen cell carrier (CCC) method. In brief, mice were anesthetized via isoflurane (2–3%). For the CCC method 4×10^4^ cells were seeded on CCC (Viscofan) one day prior to transplantation. An abdominal incision was followed by branching the portal vein using 'small artery clamps'. The left liver lobe was injured with a small superficial cut and sealed with the CCC, cell layer top down. Portal vein was unblocked and bleeding was excluded. For pain reduction all animals were treated with Rimadyl within the first 24 h upon transplantation.

After transplantation, body weights were monitored daily for a period of max. 90 days. Afterwards the animals were sacrificed; the livers were harvested from the recipient mice and immediately fixed in formalin for 24–48 h. Upon ethanol preservation, the tissues were embedded in paraffin. Then, 5 µm sections were processed that were stained against fumarylacetoacetate hydrolase (anti-rabbit FAH, Abcam; ab81087; dilution 1:50) and anti-rabbit GFP (Invitrogen; G10362; dilution 1:50). Further, the livers were examined for appearance of tumors.

Endothelial cells were transplanted using a matrigel implantation technique^28^. In brief, the spheroids with defined cell numbers (1,000 cells per spheroid; 400 spheroids per implantation) were generated using the hanging drop method with medium containing 0.25% (w/v) methylcellulose. The spheroids were harvested upon overnight cultivation. Then, 300 µl cell suspension was mixed with 300 µl matrigel (growth factor reduced, BD Biosciences) as well as fibrinogen (2 mg/ml, Calbiochem), hVEGF (1000 ng/ml, Randox), and rFGF-2 (1000 ng/ml, Bachem). Thrombin (0.4 U/µl, Calbiochem) was added to the mixture and subsequently the 600 µl matrigel-cell-suspension was subcutaneously injected into 8–12 week-old Rag2^−/−^/Il2rg^−/−^ mice. The mice were sacrificed 2–4 weeks after transplantation and the implants were dissected, fixed with paraformaldehyde (PFA) and embedded in paraffin blocks for sectioning. The sections were stained for hCD31 (Sigma; HPA004690). Tumorigenicity of cell lines was assessed by subcutaneous injection of 1×10^6^ cells into Rag2^−/−^/Il2rg^−/−^ mice and monitoring them for tumor formation for 4 months.

### Data presentation and analyses

Sample size estimation was not performed. Sample sizes in the presented experiments are similar to those used in comparable cell immortalization studies. In case n < 5, data are displayed as individual data points, otherwise sample size is stated in the figure legends. No blinding or randomization was performed and no samples or animals were excluded from analysis. Experiments were reproduced at least two times unless stated otherwise. Given the descriptive nature of this study and the sample size, no statistical tests were performed, i.e., no equal variance or normal distribution was assumed.

### Data availability

The data that support the findings of this study are available from the corresponding authors upon reasonable request. Data from gene expression analysis can be found in GEO under accession codes GSE110085 and GSE110065.

## Electronic supplementary material

Supplementary Information
