# Supplementary material for: Expansion of functional personalized cells with specific transgene combinations
Source: Nat Commun. 2018 Mar 8;9:994. doi: 10.1038/s41467-018-03408-4 (PMC5843645; doi:10.1038/s41467-018-03408-4)
Supplement: Supplementary file 1 — Supplementary Information [file 41467_2018_3408_MOESM1_ESM.pdf]

e-hChon-1

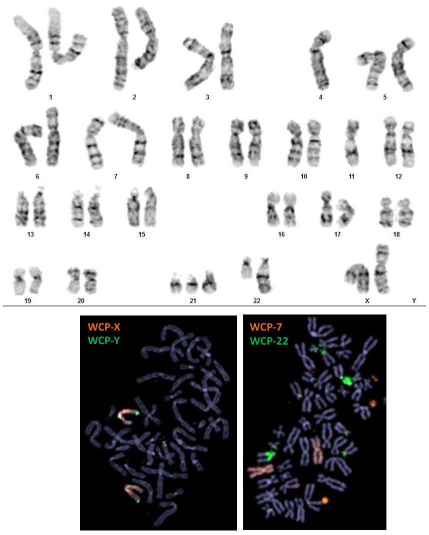

e-hDFIB2

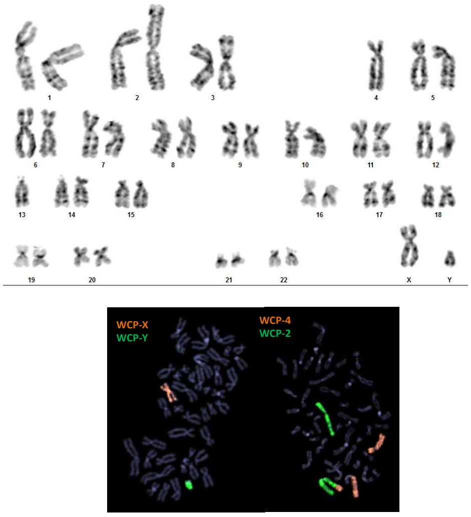

e-hFIB1

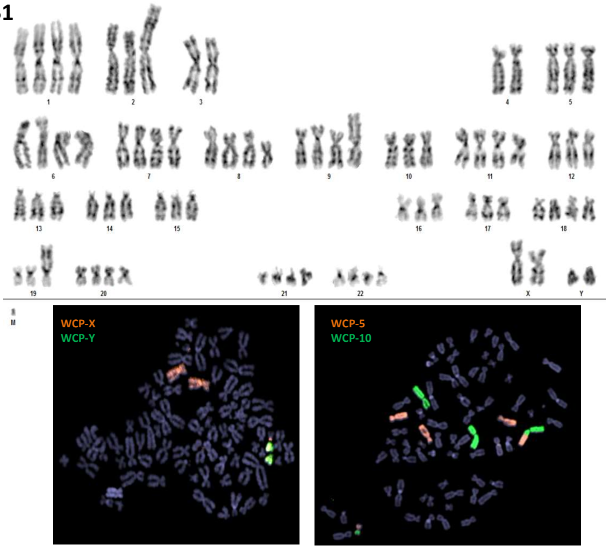

e-hOB-1

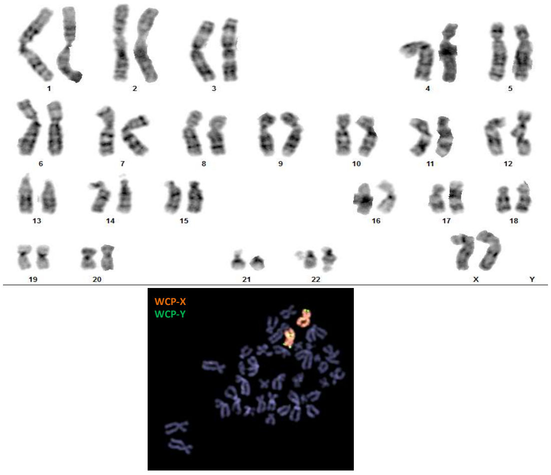
1 2 3 4 5
6 7 8 9 10 11 12
13 14 15 16 17 18
19 20 21 22 X Y
WCP-X
WCP-Y

e-hOB-2

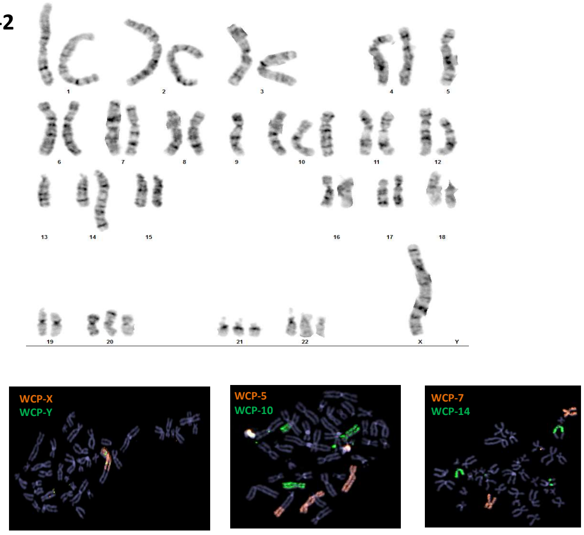
1 2 3 4 5
6 7 8 9 10 11 12
13 14 15 16 17 18
19 20 21 22 X Y
WCP-X
WCP-Y
WCP-5
WCP-10
WCP-7
WCP-14

e-hOB-3
p21

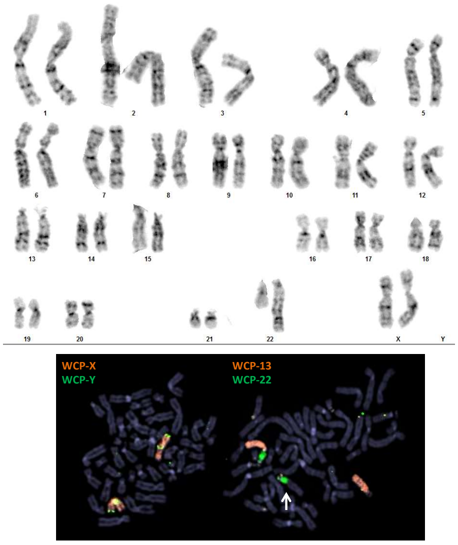

e-hOB-3
p66

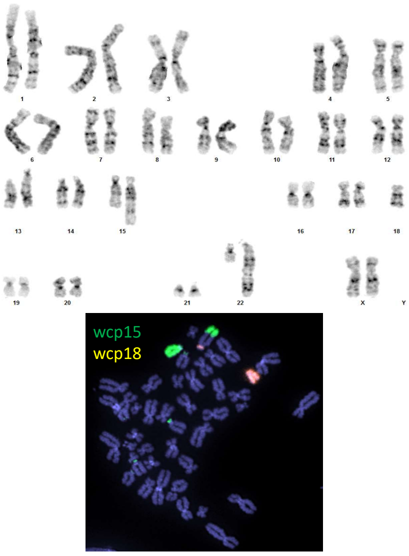

e-hStr-1

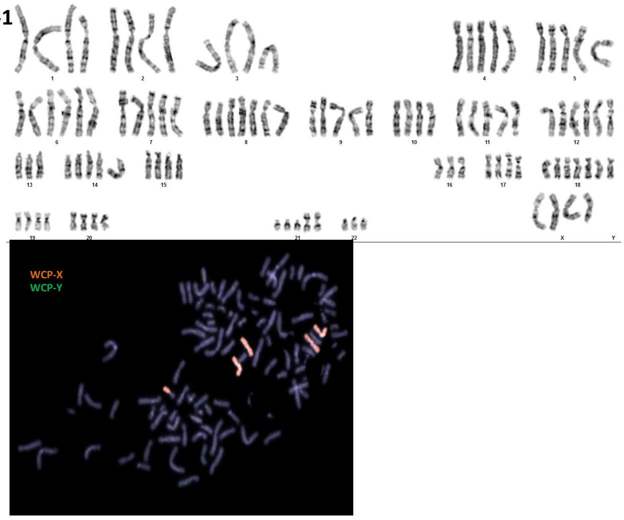

e-hStr-2

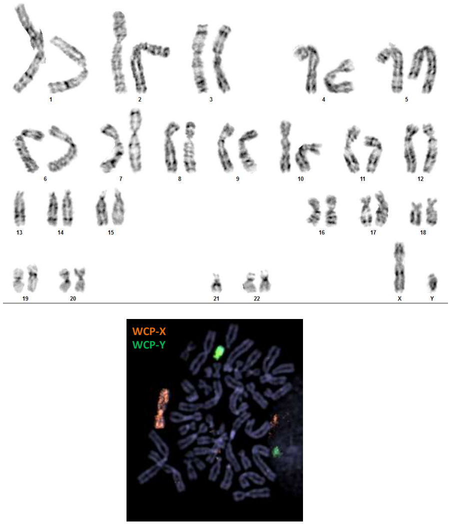

e-hUVEC-2

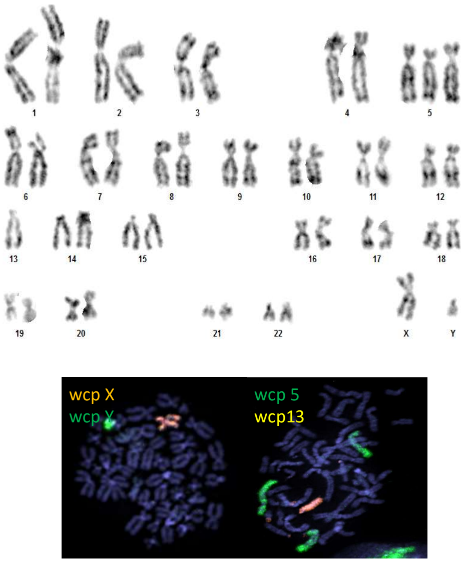

e-hUVEC-10

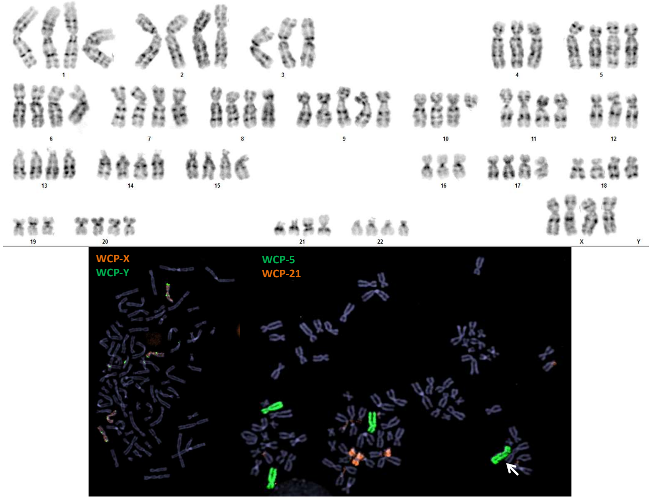

Supplementary Figure 1: Karyotype analysis of 11 expandable cell lines

Chromosome preparation and analysis have been performed according to a previously described protocol[1]. Briefly, 0.04 µM colcemid (Sigma) was added to growing cell cultures for 3 h and subjected to short hypotonic incubations (1-5 min) in 1:1 0.9% NaCitrate and 0.07M KCl and fixed by careful addition of ice-cold 3:1 methanol + acetic acid. After overnight fixation suspensions were dropped onto cold microscope slides. For G-banding slides were aged overnight at 60° C, digested in trypsin and stained with Giemsa. For whole chromosome painting directly labelled probes were used (Applied Spectral Imaging, Neckarshausen, Germany) and visualised microscopically using HiSKY software configured to an Axioimager microscope (Carl Zeiss, Jena, Germany). See Supplementary Table 2 for a summary of results.

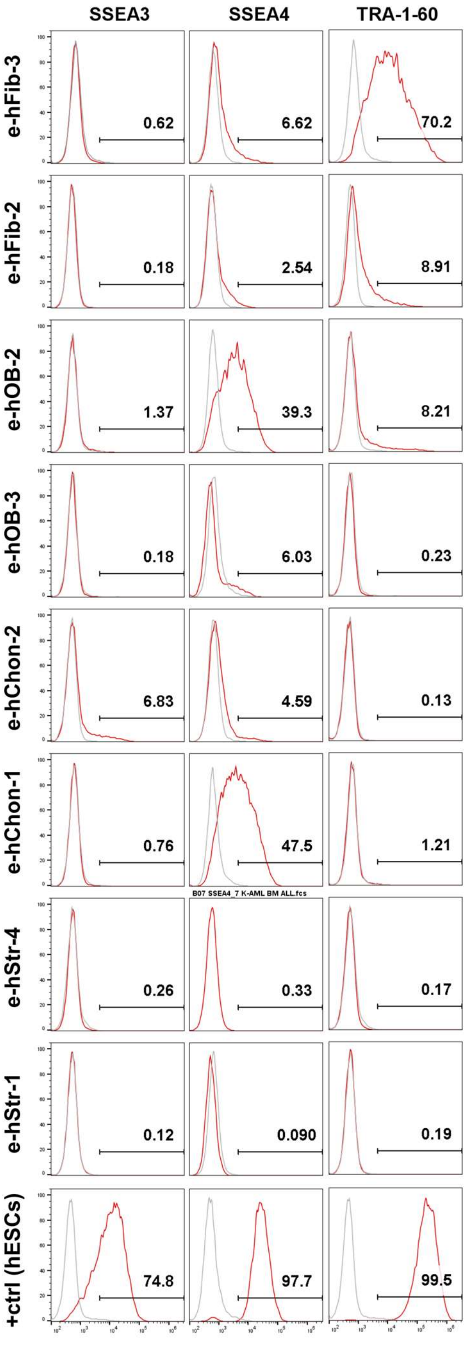

Supplementary Figure 2: Expression of pluripotency markers in novel cell lines. Flow cytometric analysis of the pluripotency-associated surface proteins SSEA-3, SSEA-4 and TRA-1-60 was performed using cell lines that incorporated pluripotency-associated genes. For this purpose, $1.5x10^6$ cells were incubated with the surface antibodies mouse anti-TRA-1-60 (1:100, Abcam), mouse anti-SSEA-3 (1:100, MC-631, Hybridoma Bank), mouse anti SSEA-4 (1:100, MC-813-70, Hybridoma Bank) and respective isotype controls (IgM and IgG3, Dako) in PBS containing 0.5% BSA for 30 minutes at 4°C. After incubation with Cy5-labeled donkey anti-mouse IgM and IgG3 antibody (1:200, Jackson ImmunoResearch), cells were analyzed

using the Accuri C6 flow cytometer (BD Biosciences). Data were processed using FlowJo (V10.1). Human embryonic stem cells (HES3) were used as positive control (+ctrl). Respective isotype controls are shown in grey. Lack of simultaneous marker expression suggests absence of pluripotency in all cell lines tested.

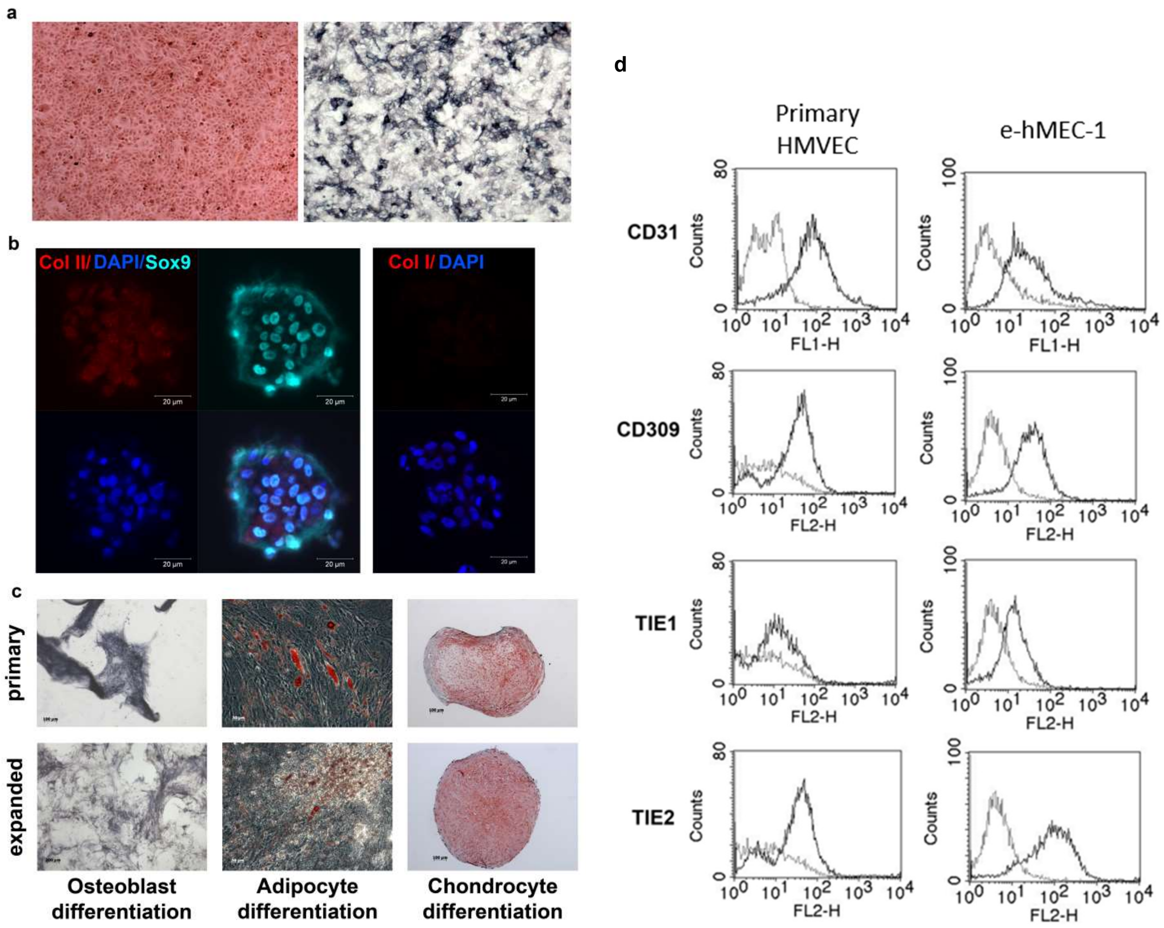

Supplementary Figure 3: Characterization of cell type specific markers. (**a**) The osteoblast cell line (e-hOB-2; *Fos, TAg, Bcl2, MYC, Nanog, EZH2,* Rex) was stained for alkaline phosphatase activity with BCIP/NBT (right) and for extracellular calcium deposits with Alizarin Red (left). For assessing the activity of the alkaline phosphatase and for determining calcium deposits osteoblast cell lines were cultivated for seven days with osteoblast differentiation media (InSCREENeX GmbH). The calcium deposits were stained with Alizarin Red S (40mM) (Sigma) after the cells were washed twice with PBS and fixed with paraformaldehyde for 5 min. The cells were stained for 15 min before they were excessively washed with water to remove remaining Alizarin Red. The alkaline phosphatase activity was determined with a BCIP/NBT substrate solution (Sigma) according to manufacturer's instructions. (**b**) Expression of chondrocyte specific markers collagen II and Sox 9 in a human chondrocyte cell line (e-hChon-3; *ID2, ID3, E6, Bcl2, core, Myc, Nanog, Sox2*) upon cultivation in spheroids as analyzed by immunofluorescence. Note that the spheroids lack collagen I expression, a dedifferentiation marker. For immunofluorescence three-dimensional chondrocyte spheroids were harvested by centrifugation at 50g for 5 min without break. Cells were fixed with ice-cold paraformaldehyde (chondrocytes) for 5 min. Primary antibodies (anti-

human collagen I (abcam cat. no. ab90395; 1:100); anti-human collagen II (abcam cat.no. ab34712; dilution 1:100); anti-human Sox9 (abcam cat.no. ab76997; dilution 1:100)) were diluted in 0.1% saponin and incubated on the washed cells for 60 min at room temperature. The secondary antibodies (goat anti-rabbit Cy3; Dianova cat. no. 111-166-045; dilution 1:100; goat anti-mouse Cy5 from Dianova cat.no. 115-175-166; dilution 1:100) were diluted in 0.1% saponin and incubated with the cells for 60 min at room temperature. Stained cells were embedded in fluoroshield (Sigma) containing DAPI. Upon incubation and drying overnight at 4°C in the dark, the stained cells were examined using the confocal microscope LSM 510 Meta (Zeiss). (**c**) The human bone marrow stroma cell line (e-hStr-3; *ID2, Fos, E7, ID1, Lmo2, Yap1, Nanog, Sox2*; lower panel) as well as primary mesenchymal stem cells (upper panel) were differentiated *in vitro* into osteoblasts, adipocytes and chondrocytes. The samples were subsequently stained for alkaline phosphatase with BCIP/NBT (left, indicating osteoblasts), for formation of lipid droplets with Oil Red (middle, indicating adipocytes) and for proteoglycan structures with SafraninO (right, indicating chondrocytes). For chondrogenic differentiation, hBMSCs derived cell lines were harvested by trypsinization. 2.5*105 cells were washed with chondrogenic differentiation medium containing high-glucose DMEM (Biochrom AG) supplemented with 40 mM HEPES (Biochrom AG), 1% penicillin (Sigma) /streptomycin (Serva), 0.1 µM dexamethasone (Calbiochem), 1 mM sodium-pyruvate (Gibco), 0.17 mM ascorbic acid-2-phosphate (Sigma), 0.35 mM proline (Sigma) and insulin-transferrin-selenium (Sigma). Then the cells were resuspended in 500 µl chondrogenic differentiation medium supplemented with 10 ng/ml TGF-β1 (R&D Systems) and spun at 350 g for 5 min. After three weeks cell pellets were fixed in 4% paraformaldehyde (Sigma) and embedded in paraffin by standard methods. To assess cartilage development, sections were stained with Alcian Blue (Sigma), Toluidin Blue (Sigma) and Safranin O (Sigma) and subjected to microscopical analysis.

To induce osteogenic development, hBMSCs derived cell lines were plated at a density of 15.000 cells/cm^3 in osteogenic differentiation medium containing low-glucose DMEM (Biochrom AG) supplemented with 10% FCS (Gibco), 40 mM HEPES (Biochrom AG), 1% penicillin (Sigma)/streptomycin (Serva), 10 mM β-glycerophosphate (Sigma), 50 µg/ml L-ascorbic acid (Sigma) and 10-7 M Dexamethasone (Calbiochem). After 3 weeks cells were fixed in 4% paraformaldehyde (Sigma) and incubated with BCIP/NBT alkaline phosphatase substrate solution (Sigma) to evaluate alkaline phosphatase activity of cultured cells.

For adipocyte differentiation, 60,000 cells/cm^3 were cultured in low-glucose DMEM (Biochrom AG) supplemented with 20% FCS (Gibco), 40 mM HEPES (Biochrom AG), 1% penicillin (Sigma) /streptomycin (Serva), 0.5 mM IBMX (Calbiochem), 60 µM indomethacin (Sigma) and 1 µM dexamethasone (Calbiochem) for 3 weeks. Staining of lipid droplets was assessed by

Oil Red (Sigma). **(d)** Characterization of novel human microvasculature endothelial cell lines. The HMVEC line (e-hMEC-1; *ID2, Fos, TAg, ID3, E7, HoxA9, ID1, MYC, Nanog, Sox2, EZH2, Gli1*) was analyzed for the expression of the endothelial specific surface markers CD31 (Pecam-1; eBioscience; cat.no. 11-0319; dilution 1:100), CD309 (VEGFR2; BD Pharmingen; cat.no. 560494; dilution 1:100), TIE1 (Abcam; cat.no. ab 27851; dilution 1:100) and TIE2 receptor (BD Pharmingen; cat.no. 557039; dilution 1:100). As control, primary HMVECs were used. The stained cells are shown in dark grey and the isotype control is shown in light grey.

a

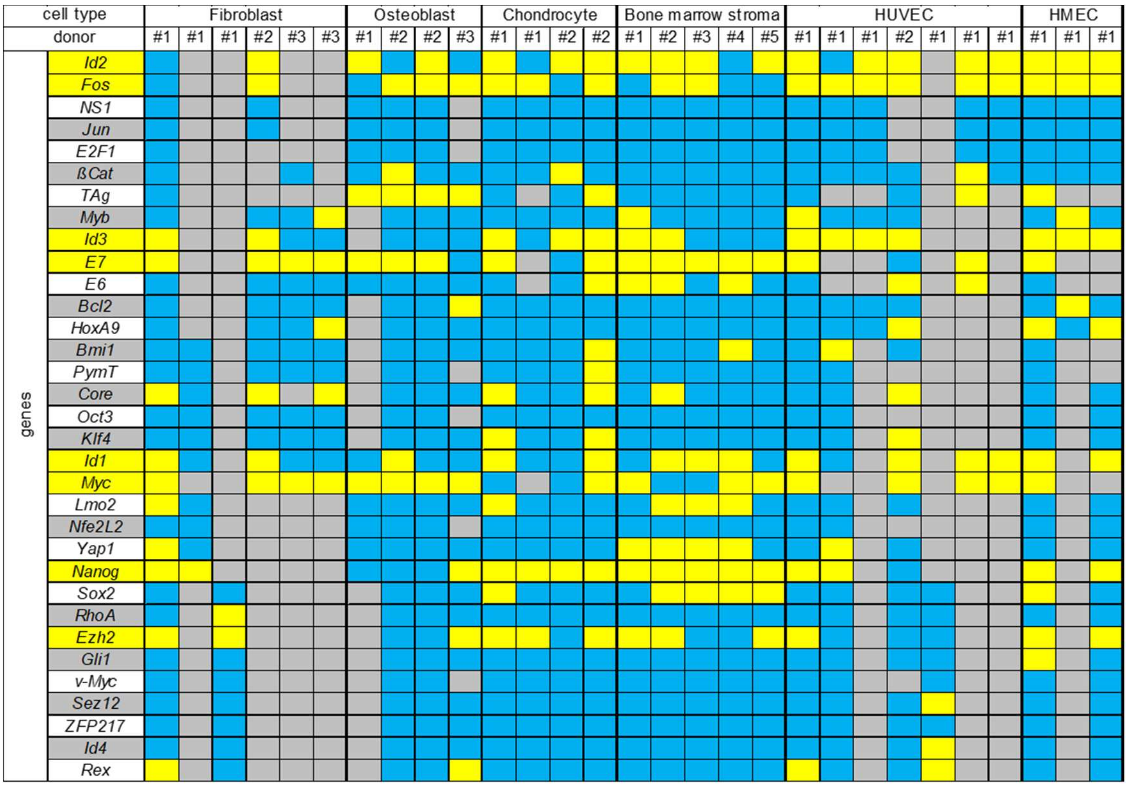

b

**Genes***	**integration† [%] n=29**
E7	**85.00**
Nanog	**84.21**
Myc	**80.95**
Id2	**78.26**
Fos	**78.26**
Ezh2	**63.16**
Id3	**60.87**
Id1	**56.00**
TAg	43.75
Core	33.33
Yap1	31.58
Sox2	31.58
E6	30.00
Lmo2	26.32
Rex	21.05
Myb	17.39
HoxA9	17.39
Bmi1	14.29
Klf4	13.64
ßCat	13.04

*Only those genes that were detected in at least 10% of the analyzed cell lines are shown.

† Most abundant genes identified in 29 cell lines of various cell sources (see text for further explanations).

Supplementary Figure 4: Chromosomal integration of library genes in novel human cell lines from different cell types. (a) Various cell lines from primary human cell types (foreskin fibroblasts (#1 and #2); dermal fibroblasts (#3), osteoblasts; chondrocytes, bone marrow stroma cells, HUVEC, HMEC) of different donors were analyzed for the integrated library genes by PCR. Yellow: gene integrated; blue: not integrated; grey: gene not used in the respective infection. Key set of genes is highlighted in yellow. **(b)** Integration frequency of library genes in novel human cell lines.

a

b

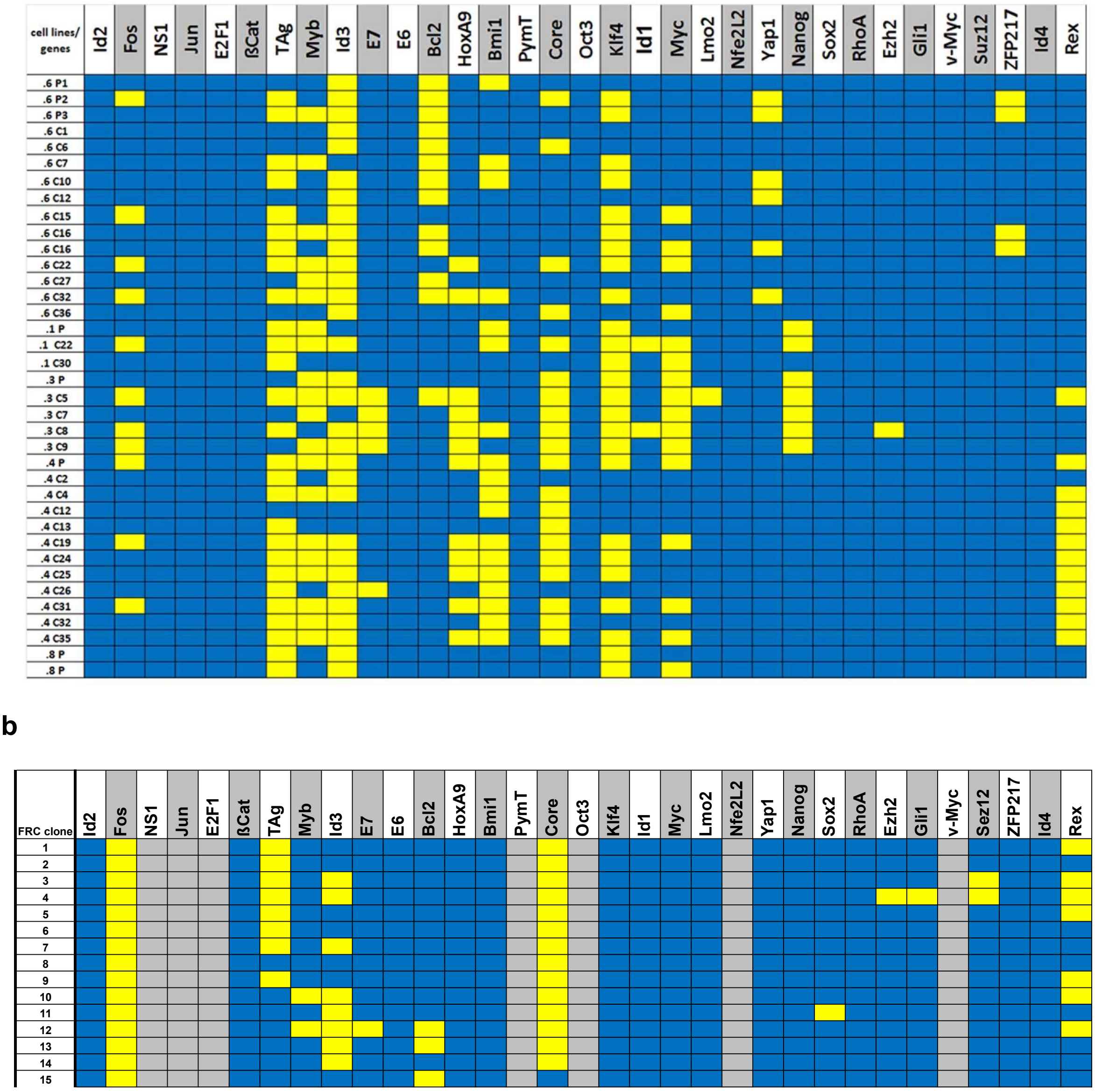

Supplementary Figure 5. Chromosomal integration of library genes in novel cell lines. Genes integrated into **(a)** hepatocytes and **(b)** fibroblastoid reticular cells (FRCs). Yellow: gene integrated; blue: not integrated; grey: gene not used in the respective infection

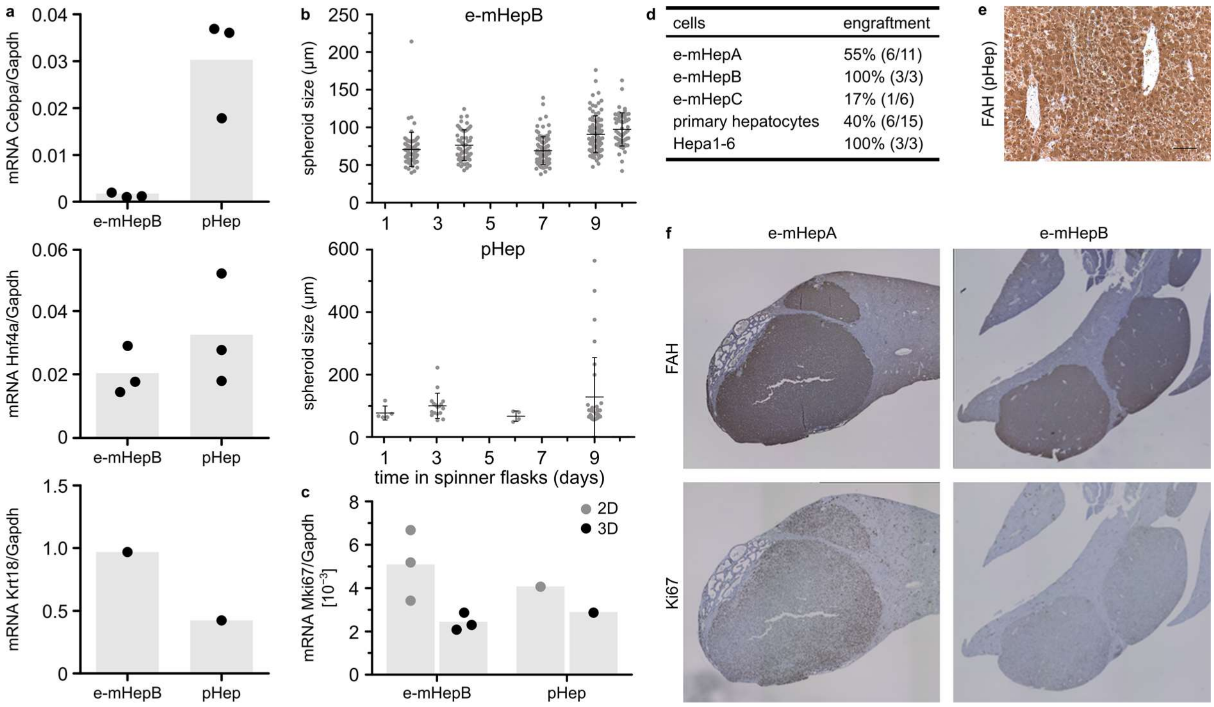

Supplementary Figure 6: **Characterization of murine hepatocyte cell lines.** (**a**) Expandable hepatocytes (e-mHepB) and primary murine hepatocytes (pHep) were characterized by qRT-PCR. The expression level of the hepatic markers HNF4α, CK18 and C/EBPα was related to Gapdh. Bars represent mean. (n=3 independent experiments for Cebpa and Hnf4a, n = 1 for Krt18). (**b**) Cell line e-mHepB and freshly isolated primary hepatocytes were transferred to spinner flasks for 3D cultivation to establish spheroids. The diameter of single spheroids was regularly monitored by phase contrast microscopy. Error bars represent mean ± standard deviation. (**c**) The expression of the proliferation marker Ki67 was analyzed by qRT-PCR in expanded hepatocytes that were cultivated in monolayer cultures (2D) and in spinner cultures (3D) for nine days. As control, Ki67 levels are depicted in primary hepatocytes directly after isolation (fresh) or after nine days of 3D culture. Bars represent mean (n=3 independent experiments for e-mHepB, n = 1 for primary hepatocytes). (**d**) Hepatic cell lines with the indicated integrated genes and primary murine hepatocytes were engrafted into FRG mice by intrasplenic injection ($2x10^6$ cells) or transfer via collagen-coated carriers ($2x10^4$ cells). Cells were considered to have engrafted when FAH positively-stained islets were observed after immunohistochemical analysis of livers. **(e)** As a control for the experiment shown in Figure 2g, primary murine hepatocytes were transplanted into FRG mice. After three months animals were sacrificed and livers were stained for FAH, Scale bar, 100 µm. **(f)** Immunohistochemistry shows islets of Ki67 positive cells after engraftment of cell lines into the liver parenchyma of FRG mice. Livers of animal were analyzed 90 days post cell transplantation.

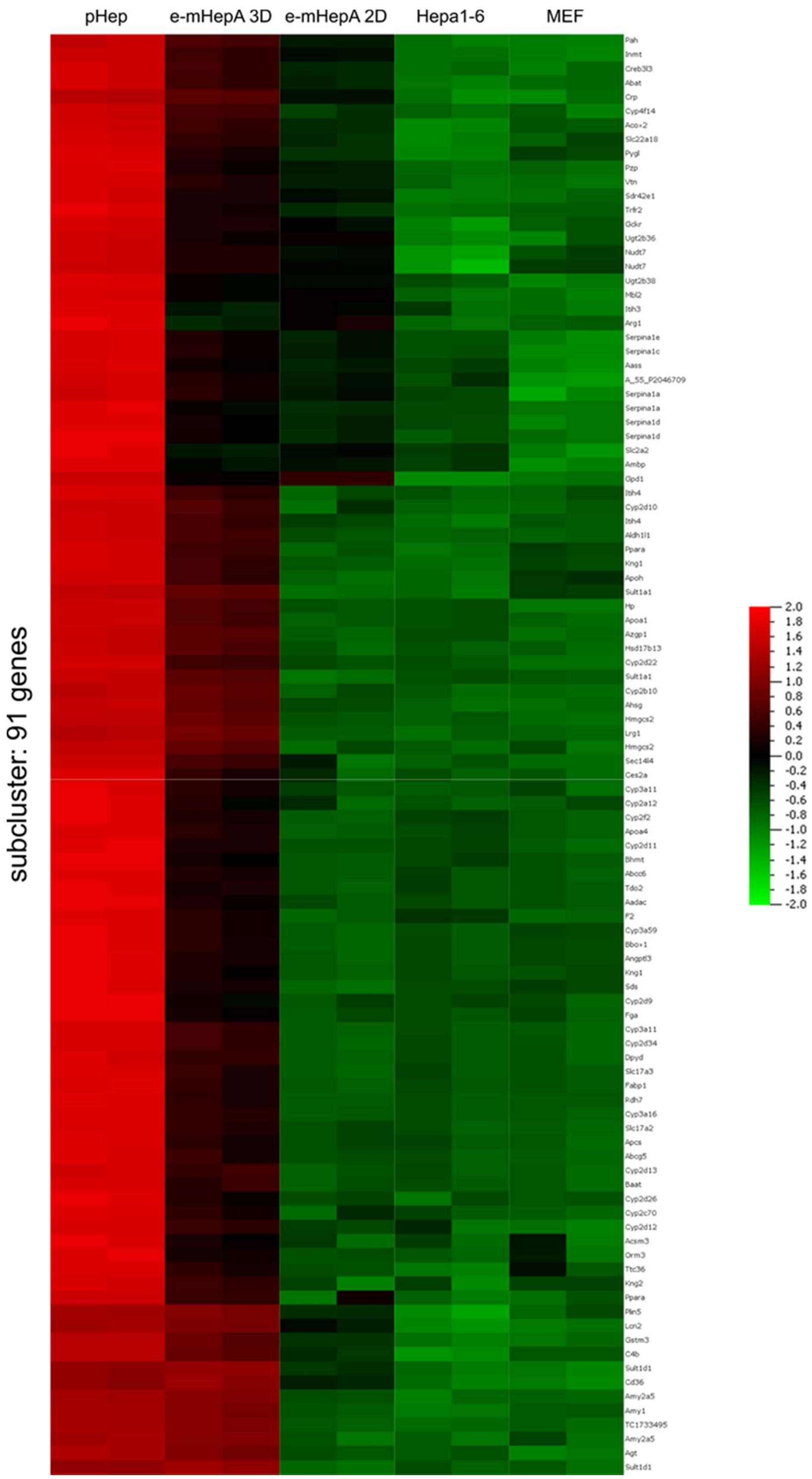

Supplementary Figure 7: Microarray gene expression analysis of hepatocytes. A subcluster containing upregulated genes in expanded hepatocytes upon cultivation in 3D is

shown. Global gene expression was analyzed in freshly isolated murine primary hepatocytes (pHep, n=2), the e-mHepA cell line cultivated in 2D (n=2) and 3D (n=2). As controls the hepatocellular carcinoma cell line Hepa1-6 (n=2) and mouse embryonic fibroblasts (MEF, n=2) were included. Microarray expression data were imported into Omics Explorer software v3.0 (Qlucore) for hierarchical cluster analysis and heatmap visualization using default import settings for Agilent single color microarrays. A subcluster of 102 variables/ 91 genes with prominent gene expression signatures was identified upon filtering datasets with a 0.40 projection score corresponding to the removal of the lowest 50pc of overall variance. This cluster contains major factors that are involved in the drug metabolism like phase I enzymes of the Cyp450 family as well as phase 2 enzymes such as Sult1d1 and Sult1a1.

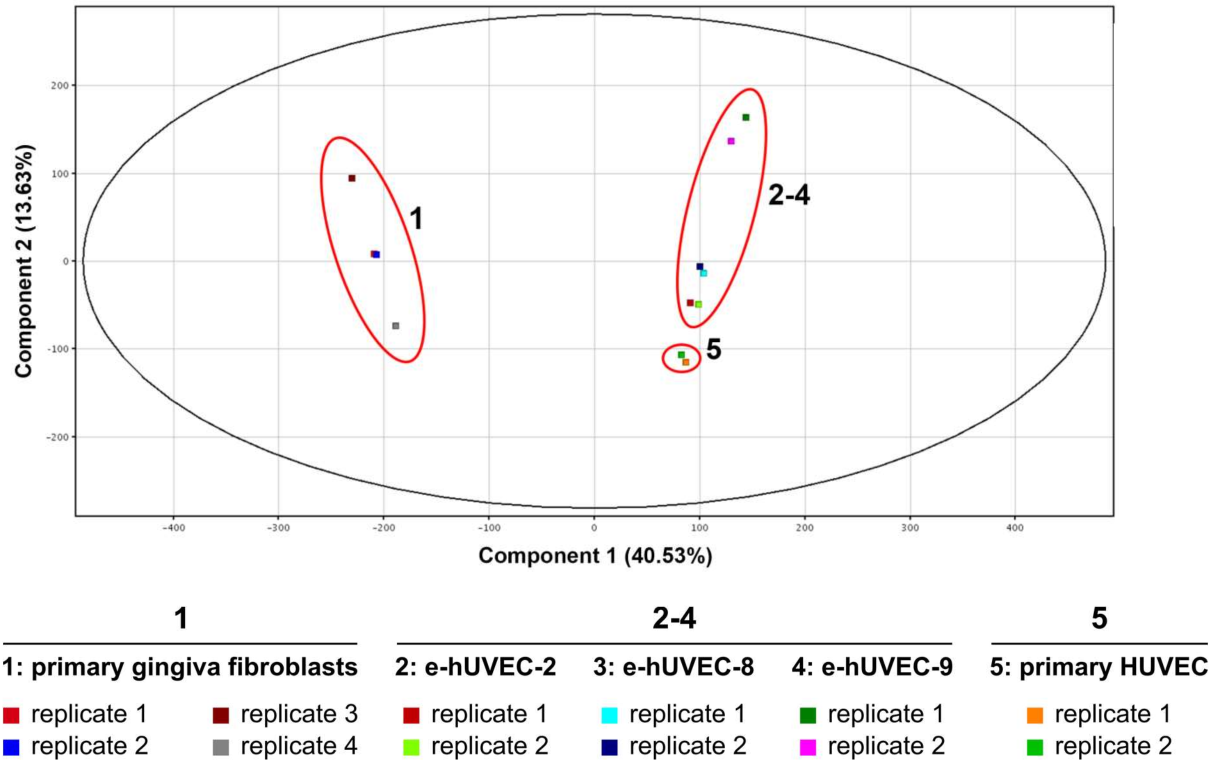

Supplementary Figure 8: Correlation analysis of global gene HUVEC expression by PCA. Global gene expression was analyzed for two independent primary and three HUVEC lines (e-hUVEC-2; e-hUVEC-8, e-hUVEC-9) as well as primary gingiva fibroblasts. The microarray data were processed by the GeneSpring software to classify the different cell samples by principal component analysis (PCA). Each dot represents one sample of a cell line or of primary cells. The first two principal components (PC) of the PCA mapping account for 54.16% variance (PC1: 40.53%, PC2: 13.63%) of the data set. The HUVEC lines are arranged in a cluster together with their primary counterparts and are clearly separated from the fibroblasts.

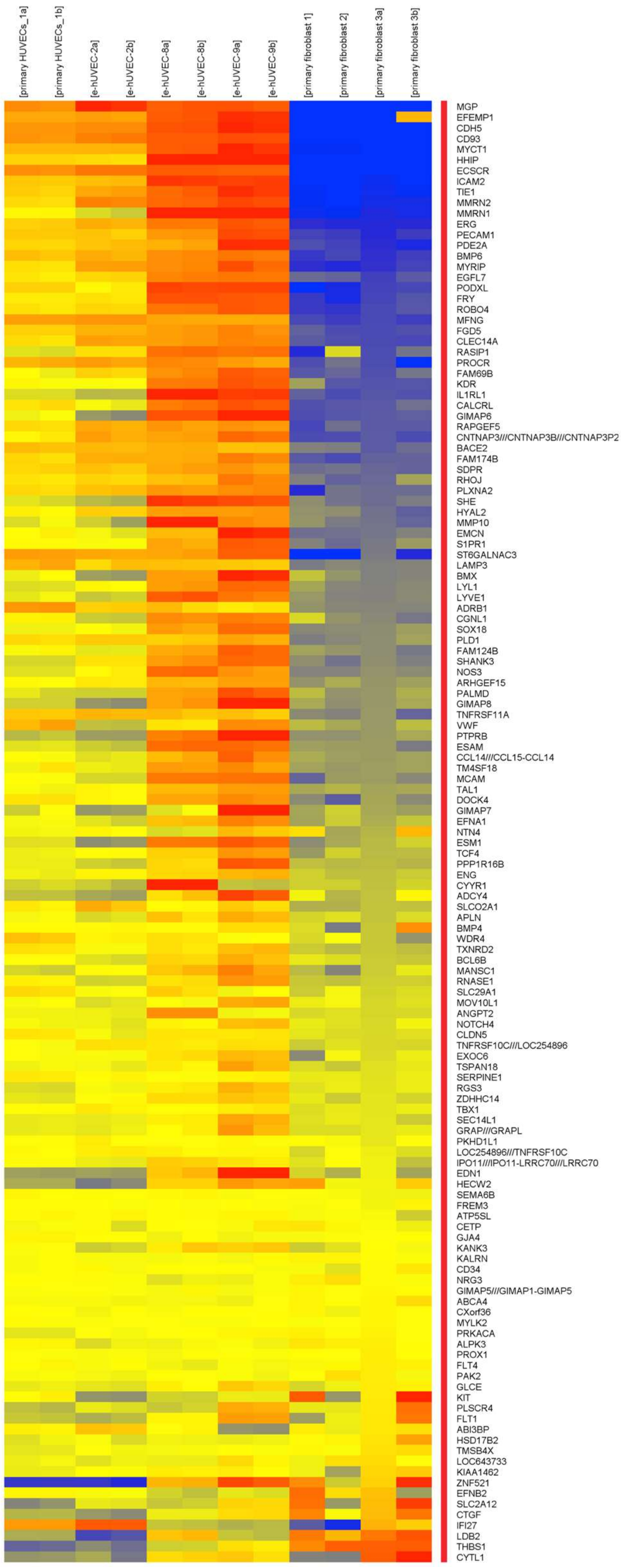

Supplementary Figure 9. Microarray gene expression analysis of endothelial specific genes. Analysis of endothelial genes (see main text for details and reference) in primary and immortalized cell lines e-hUVEC-2, e-hUVEC-8, e-hUVEC-9. Three primary human fibroblast cultures are included as control (primary fibroblasts 1-3, fibroblast 3 in early (3a) and late (3b) culture. Analysis of two replica per endothelial cell sample is shown (designated as a and b), a single sample is shown for the fibroblasts.

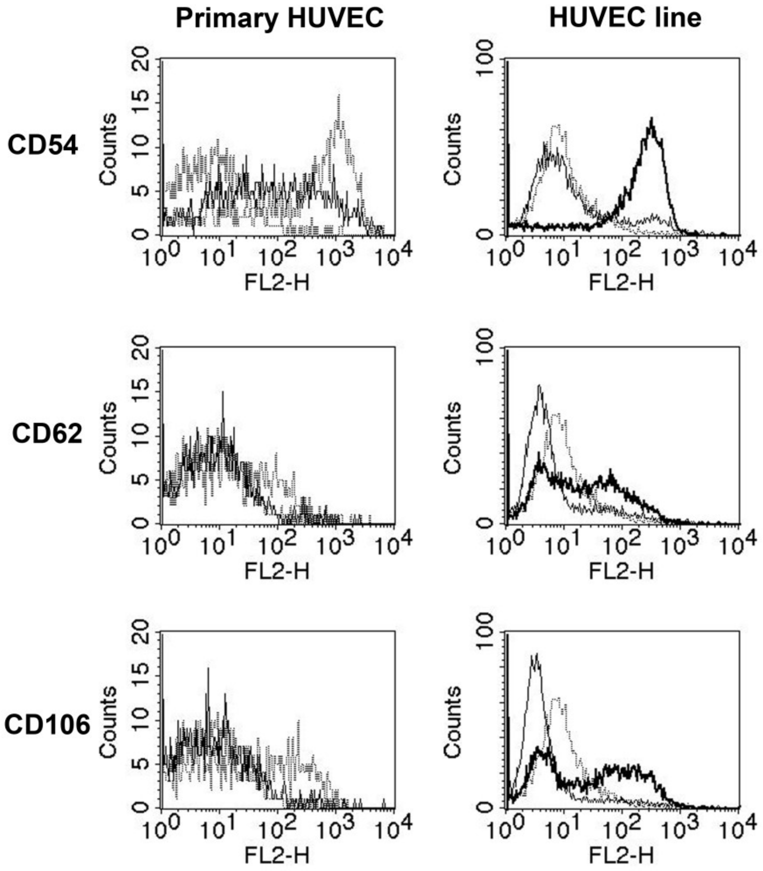

Supplementary Figure 10: Novel HUVEC lines respond to TNFα. For the analysis of the TNFα inducible genes the cells of a HUVEC line (e-hUVEC-2; *MYC, ID1, ID2*) were incubated for four hours with TNFα (25 ng/ml). Flow cytometry was performed to assess expression of CD54 (ICAM1; eBioscience; cat.no. 12-0549; dilution 1:100), CD62E (E-Selectin; eBioscience; 12-0627; dilution 1:100), and CD106 (VCAM; eBioscience; cat.no. 12-1069; dilution 1:100) upon staining with fluorescently labeled antibodies. Isotype control: light grey; Antibody stained cells without TNFα: dark grey; Antibody stained cells treated with TNFα: black.

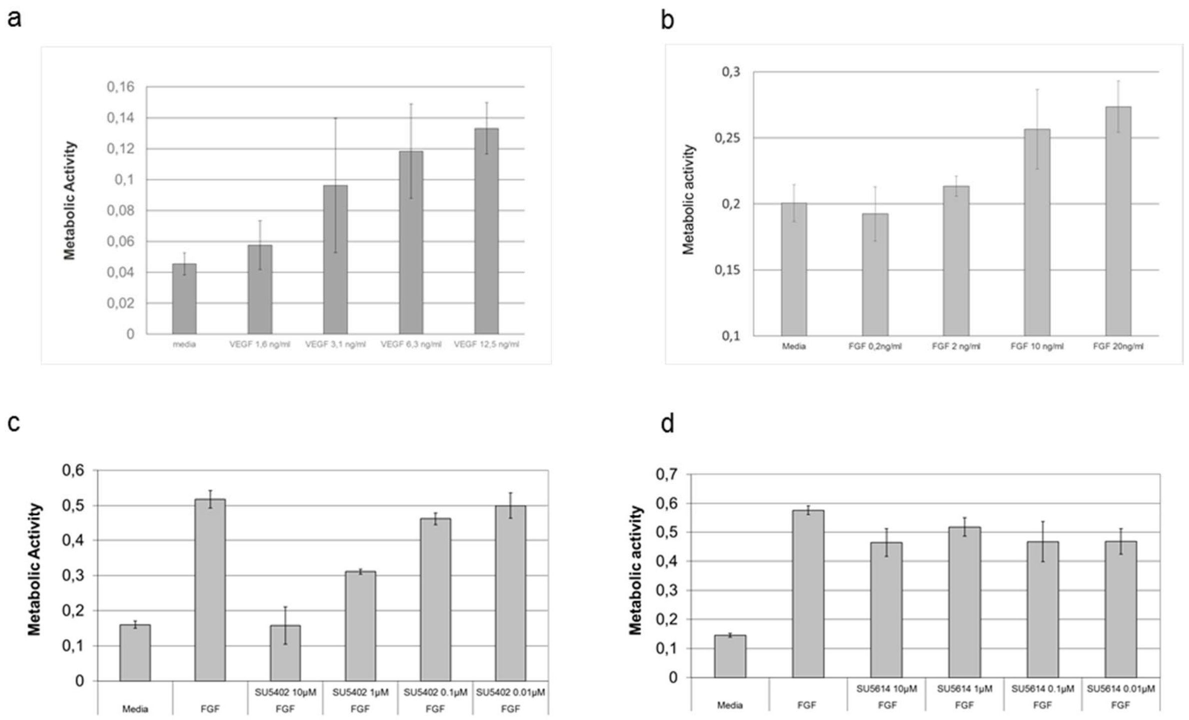

Supplementary Figure 11: Modulation of proliferation by cytokine signaling. The effect of the different cytokines on the proliferation of the HUVEC line (e-hUVEC-2; *MYC, ID1, ID2*) was determined by cultivating $1*10^4$ cells for three days in presence of increasing concentrations of (**a**) VEGF and (**b**) FGF (both Reliatech, Germany) in 96 well format. To confirm specificity, (**c**) an FGF inhibitor (SU5402) and (**d**) an irrelevant VEGF inhibitor (SU 5614) were added to the culture (Merck Millipore, Germany). Proliferation was quantified after three days by determining spectrophotmetrically the metabolic activity of the cells (mean values of n=8 technical replicates as well as standard deviation are provided) with WST-1 (Roche, Germany) reagent according to manufacturer’s instructions.

Supplementary Table 1: Library of 33 genes used for the generation of cell lines from primary cells

		gene title	gene symbol	GeneID	species	Sequence ID
1	Id2	inhibitor of DNA binding 2 (Id2)	ID2	GeneID: 3398	human	NM_002166.4|
2	Fos	v-fos FBJ murine osteosarcoma viral oncogene homolog	FOS	GENE ID: 2353	human	NM_005252.2|
3	NS1	NS1 nonstructural protein NS1	NS1	GeneID: 956533	Influenza Virus	CY044313.1|
4	Jun	jun oncogene	JUN	GENE ID: 3725	human	NM_002228.3|
5	E2F1	Homo sapiens E2F transcription factor 1 (E2F1)	E2F1	GENE ID: 1869	human	NM_005225.1|
6	ßCat	CTNNB1 catenin (cadherin-associated protein), beta 1, 88kDa	CTNNB1	GeneID: 1499	human	NM_001098209.1
7	TAg	SV40gp6 large T antigen	SV40gp6	GeneID: 1489531	Simian Virus 40	
8	Myb	v-myb myeloblastosis viral oncogene homolog (avian)	MYB	GENE ID: 4602	human	NM_005375.2
9	Id3	inhibitor of DNA binding 3, dominant negative helix-loop-helix protein	ID3	GENE ID: 3399	human	NM_002167.3
10	E7	E7 transforming protein	E7	GeneID: 1489079	Human papillomavirus type 16	
11	E6	E6 transforming protein	E6	GeneID: 1489078	Human papillomavirus type 16	
12	Bcl2	B-cell leukemia/lymphoma 2	Bcl2	GENE ID: 12043	murine	NM_009741.3
13	HoxA9	homeobox A9	HOXA9	GENE ID: 3205	human	NM_152739.3
14	Bmi1	Bmi1 polycomb ring finger oncogene	Bmi1	GeneID: 12151	murine	NM_007552.4
15	PymT	MPyVgp2 middle t-antigen	MPyVgp2	GeneID: 1489533	Polyoma Virus	
16	Core	core protein			Hepatitis C Virus	
17	Oct3	POU class 5 homeobox 1	POU5F1	GENE ID: 5460	human	NM_002701.4
18	Klf4	Kruppel-like factor 4	KLF4	GeneID: 9314	human	NM_004235.3
19	Id1	inhibitor of DNA binding 1, dominant negative helix-loop-helix protein	ID1	GENE ID: 3397	human	NM_002165.2
20	Myc	v-myc myelocytomatosis viral oncogene homolog (avian)	MYC	GeneID: 4609	human	NM_002467.4
21	Lmo2	LIM domain only 2 (Lmo2),	Lmo2	GeneID: 4005	murine	NM_008505.3
22	Nfe2L2	nuclear factor (erythroid-derived 2)-like 2	NFE2L2	GENE ID: 4780	human	NM_006164.2|
23	Yap1	yes-associated protein 1	Yap1	GENE ID: 22601	murine	NM_009534.2|
24	Nanog	Nanog homeobox (Nanog)	Nanog	GeneID: 71950	murine	NM_028016.1|
25	Sox2	SRY (sex determining region Y)-box 2	SOX2	GENE ID: 6657	human	NM_003106.2
26	RhoA	ras homolog gene family, member A	RHOA	GeneID: 387	human	BC001360, BC001360.2, BE304744, BE304744.1
27	Ezh2	enhancer of zeste homolog 2 (Drosophila)	EZH2	GeneID: 2146	human	BC010858, BC010858.2, BE905143, BE905143.1
28	Gli1	glioma-associated oncogene homolog 1 (zinc finger protein)	GLI1	GENE ID: 2735	human	NM_005269.1|
29	v-Myc	v-myc myelocytomatosis viral related oncogene, neuroblastoma derived	MYCN	GeneID: 4613	human	BC002712, BC002712.2, BE382701, BE382701.1
30	Suz12	suppressor of zeste 12 homolog	SUZ12	GeneID: 23512	human	BC015704, BC015704.1, BE887049, BE887049.1
31	ZFP217	zinc finger protein 217	ZNF217	GeneID: 7764	human	BC113427, BC113427.1
32	Id4	inhibitor of DNA binding 4, dominant negative helix-loop-helix protein	ID4	GENE ID: 3400	human	NM_001546.2
33	Rex	Mus musculus zinc finger protein 42 (Zfp42)	Zfp42	GeneID: 22702	murine	NM_009556.2

Supplementary Table 2: Summary of cytogenetic analyses

Cell line	Karyotype	ploidy	Structural rearrange-ments
e-hChon-1	46<2n>XX,del(2)(q3?),-4,add(7)(p14),+21,add(22)(q13).	2N	3
e-hDFIb2	44<2n>XY, der(2)t(2;4)(p24;p11),-4,-13, add(14)(p11).	2N	2
e-hFib1	78<3n>XXYY,+1,add(2)(p24),-3,+6,+7,+del(8)(q21),+9,add(9)(p2?),+10, der(10)t(5;10)(q15:p15),+11,+18,der(19)t(11;19)(q13;p13),+20,+21,+22,+2mar.	3N	7
e-hFib2	69<3n>XXYY,+X/Y, t(2;5)(p16;q13),+3,+5,-9,+11,-13,+16,+20,-21,-22.	3N	1
e-hOB-1	46<2n>XX.	2N	0
e-hOB-2	40-47<2n>add(X)q27),add(10)(p11),-13,add(14)(q32),+20,+21,+22.	2N	3
e-hOB-3 p21	46<2n>XX, add(22)(q13).	2N	1
e-hOB-3 p66	46<2n>XX, ins (15)(q11),add(22)(q13).	2N	2
e-hStr-1	111<5n>XXXX,-X,del(X)(q25),del(1)(p22),i(21)(q10).	5N	3
e-hStr-2	44<2n>XY,der(5)t(5;14)(p12;q11),-13,-21.	2N	1
e-hUVEC-10	84-88<4n>XXXX,-3,-4,+9,i(21)(q10)	4N	1
e-hUVEC-2	46<2n>XY, +5,-13	2N	0

Supplementary Table 2 shows 12 consensus karyotypes of 11 cell lines, including serial samples of e-hOB-3 at passages 21 and 66, together with reference ploidies and numbers of rearranged chromosomes detected.

Supplementary Table 3: Primer sequences applied for the identification of chromosomally integrated library genes

Primer	Sequence
SV40for1	GGAGGCCTAGGCTTTTGCAA
Id2	GCAGGCTGACAATAGTGGGA
Fos	GGATGATGCTGGGAACAGGA
NS1	ATGTCCTGGAAGAGAAGGCA
Jun	TTCCTCATGCGCTTCCTCTC
E2F1	CAGGGTCTGCAATGCTACGA
ßCat	TTATGCAAGGTCCCAGCGGT
TAg	CACCTGGCAAACTTTCCTCA
Myb	CTTCTGGAAGCTTGTGGCCA
Id3	ATGACAAGTTCCGGAGCGAG
E7	GCCCATTAACAGGTCTTCCA
E6	ATTCGCCCTTTTACAGCTGG
Bcl2	TCTGCGAAGTCACGACGGTA
HoxA9	GTTTAATGCCATAAGGCCGG
Bmi1	GGGCCATTTCTTCTCCAGGT
PymT	CATCTCGGGTTGGTGTTCCA
Core	ACTTTACCCACGTTGCGCGA
Oct3	GCAAAGCAGAAACCCTCGTG
Klf4	AAGATCAAGCAGGAGGCGGT
Id1	AGAAGCACCAAACGTGACCA
Myc	AGTGGGCTGTGAGGAGGTTT
Lmo2	TTTCCGTCCCAGCTTGTAGT
Nfe2L2	GCTGCTGAAGGAATCCTCAA
Yap1	GCCAGGATGTGGTCTTGTTC

Nanog	TATGGAGCGGAGCAGCATTC
Sox2	CTCGCAGACCTACATGAACG
RhoA	AAGCATTTCTGTCCCAACGT
Ezh2	ACTTCGAGCTCCTCTGAAGC
Gli1	CACCACATCAACAGCGAGCA
v-Myc	GACACCCTGAGCGATTCAGA
Sez12	TACCCTGGAAGTCCTGCTTG
ZFP217	CAAGAAGGGAGCACCGACAA
Id4	CAGCAAAGTGGAGATCCTGC
Rex	GCGAGCTCATTACTTGCAGG
Id2	GCAGGCTGACAATAGTGGGA
Fos	GGATGATGCTGGGAACAGGA
NS1	ATGTCCTGGAAGAGAAGGCA

Supplementary Table 4: Primer sequences used for qRT-PCR[2]

Primer name	Sequence
Gapdhfwd	CCTGCACCACCAACTGCTTA
Gapdhrev	TCAATGAGCCCCTTCCACAATG
Albfwd	CTCAGGTGTCAACCCCAA
Albrev	TCCACACAAGGCAGTCTC
CK18fwd	CGAGGCACTCAAGGAAGAAC
CK18rev	CTTGGTGGTGACAACTGTGG
CEBPafwd	AAGAAGTCGGTGGACAAGAACAG
CEBParev	GTTGCGTTGTTTGGCTTTATCTC
HNF4afwd	TGCCAACCTCAATTCATCCA
HNF4arev	GCTCGAGGCTCCGTAGTGTT
G6pcfwd	TCTGTCCCGGATCTACCTTG
G6pcrev	GTAGAATCCAAGCGCGAAAC
Ki67	Mm_Mki67_1_SG QuantiTect Primer Assay (Qiagen)
Cyp1a1fwd	GGTTAACCATGACCGGGAACT
Cyp1a1rev	TGCCCAAACCAAAGAGAGTGA
Cyp3a11fwd	CAGCTTGGTGCTCCTCTACC
Cyp3a11rev	TCAAACAACCCCCATGTTTT

Supplementary References

1. MacLeod, R.A., Kaufmann, M.E. & Drexler, H.G. Cytogenetic Harvesting of Cancer Cells and Cell Lines. Methods in molecular biology (Clifton, N.J 1541, 43-58 (2017).

2. Iacob, R., et al. Induction of a mature hepatocyte phenotype in adult liver derived progenitor cells by ectopic expression of transcription factors. Stem cell research 6, 251-261 (2011).
